# Spontaneously slow-cycling subpopulations of human cells originate from activation of stress-response pathways

**DOI:** 10.1371/journal.pbio.3000178

**Published:** 2019-03-13

**Authors:** Mingwei Min, Sabrina L. Spencer

**Affiliations:** Department of Biochemistry and BioFrontiers Institute, University of Colorado-Boulder, Boulder, Colorado, United States of America; UCLA, UNITED STATES

## Abstract

Slow-cycling subpopulations exist in bacteria, yeast, and mammalian systems. In the case of cancer, slow-cycling subpopulations have been proposed to give rise to drug resistance. However, the origin of slow-cycling human cells is poorly studied, in large part due to lack of markers to identify these rare cells. Slow-cycling cells pass through a noncycling period marked by low CDK2 activity and high p21 levels. Here, we use this knowledge to isolate these naturally slow-cycling cells from a heterogeneous population and perform RNA sequencing to delineate the transcriptome underlying the slow-cycling state. We show that cellular stress responses—the p53 transcriptional response and the integrated stress response (ISR)—are the most salient causes of spontaneous entry into the slow-cycling state. Finally, we show that cells’ ability to enter the slow-cycling state enhances their survival in stressful conditions. Thus, the slow-cycling state is hardwired to stress responses to promote cellular survival in unpredictable environments.

## Abstract 2

Single-cell time-lapse imaging and transcriptomic analysis reveal why some human cells proliferate more slowly than the majority of the population, with cellular stress responses triggering entry into a slow-cycling state to promote cellular survival in unpredictable environments.

## Author summary

Even within a genetically identical population, some cells proliferate more slowly than others. Slow-cycling cells have been implicated in resistance to antibiotics, antifungals, and cancer therapies, yet the origin of the slow-cycling state remains poorly understood. Here, we isolate a naturally slow-cycling subpopulation of human cells and find that the slow-cycling state is induced by moderate activation of stress responses. We further show that the ability to enter this slow-cycling state protects cells from further stress, consistent with its association with drug resistance. We propose that the existence of the slow-cycling state thereby promotes long-term survival of populations that occasionally experience mildly stressful environments.

## Introduction

From an evolutionary perspective, individuals that give rise to the highest number of progeny are considered to be the fittest. However, proliferation rate is often highly variable, even in a genetically identical population in optimal growth conditions. This heterogeneity is marked by the presence of a small population of slow-cycling cells observed in bacteria [1], yeast [2], and human cells [3–5]. In unicellular organisms such as bacteria and yeast, this heterogeneity in proliferation rate has been proposed to serve as a bet-hedging mechanism, in which the slow-cycling subpopulation can be better suited to tolerate harsh conditions, giving rise to increased fitness in a variable environment in the long term [6]. The long-term benefit allows the heterogeneity itself to be selected as a conserved trait.

In slow-cycling cells, the relatively long time between two cell division events is often attributed to a prolonged noncycling state that precedes commitment to the cell cycle [7–9]. In multiple primary, immortalized but non-transformed, and cancerous human cells, we previously reported that a majority of cells commits to another cell cycle soon after mitosis, marked by increasing CDK2 activity and hyperphosphorylated Rb (CDK2^inc^ cells), while a separate subpopulation enters a transient G0/quiescence marked by low CDK2 activity, hypophosphorylated Rb, and declining Ki67 levels (CDK2^low^ cells) [10–12]. These cells can later re-enter the cell cycle by increasing CDK2 activity, indicating the reversibility of this state [10,13]. We refer to this unprompted entry into the CDK2^low^ state as “spontaneous G0” or “spontaneous quiescence,” to contrast with canonical quiescent states, in which cells are forced into quiescence by serum starvation or contact inhibition [14].

We and others have recently shown that most CDK2^low^ cells express high levels of a cyclin-dependent kinase (CDK) inhibitor, p21, which is the dominant cause of entry into the spontaneous CDK2^low^ state [15–17]. About 50% of these CDK2^low^ cells harbor low levels of endogenous DNA damage, marked by the presence of 53BP1 nuclear bodies and γH2AX foci, which triggers p53-mediated up-regulation of p21 [15,16]. However, the trigger for entry into the CDK2^low^ slow-cycling state in the other 50% of CDK2^low^ cells remains unknown.

In this study, we isolate spontaneous CDK2^low^ cells from normally cycling cells and characterize the transcriptome of this subpopulation of slow-cycling cells. We first show that the slow-cycling state is a long-lived but reversible state. Transcriptomic analysis reveals a stress-response signature in the spontaneous CDK2^low^ subpopulation, which is not present in cells forced into quiescence using four other methods. More specifically, we detect a strong signature of a p53 transcriptional program as well as activation of the integrated stress response (ISR). Knockout of p53 or its transcriptional target p21 eliminates the spontaneous CDK2^low^ subpopulation, giving rise to a more homogenous fast-cycling population. However, such cells that are unable to enter the spontaneous CDK2^low^ state are quite vulnerable to exogenous stress, manifesting as a dramatic drop in fitness in stressful environments. Our data suggest that entering a CDK2^low^ quiescence is an important mechanism to protect cells from stress.

## Results

### Isolation of the slow-cycling subpopulation and characterization of its longevity

**Fig 1:**
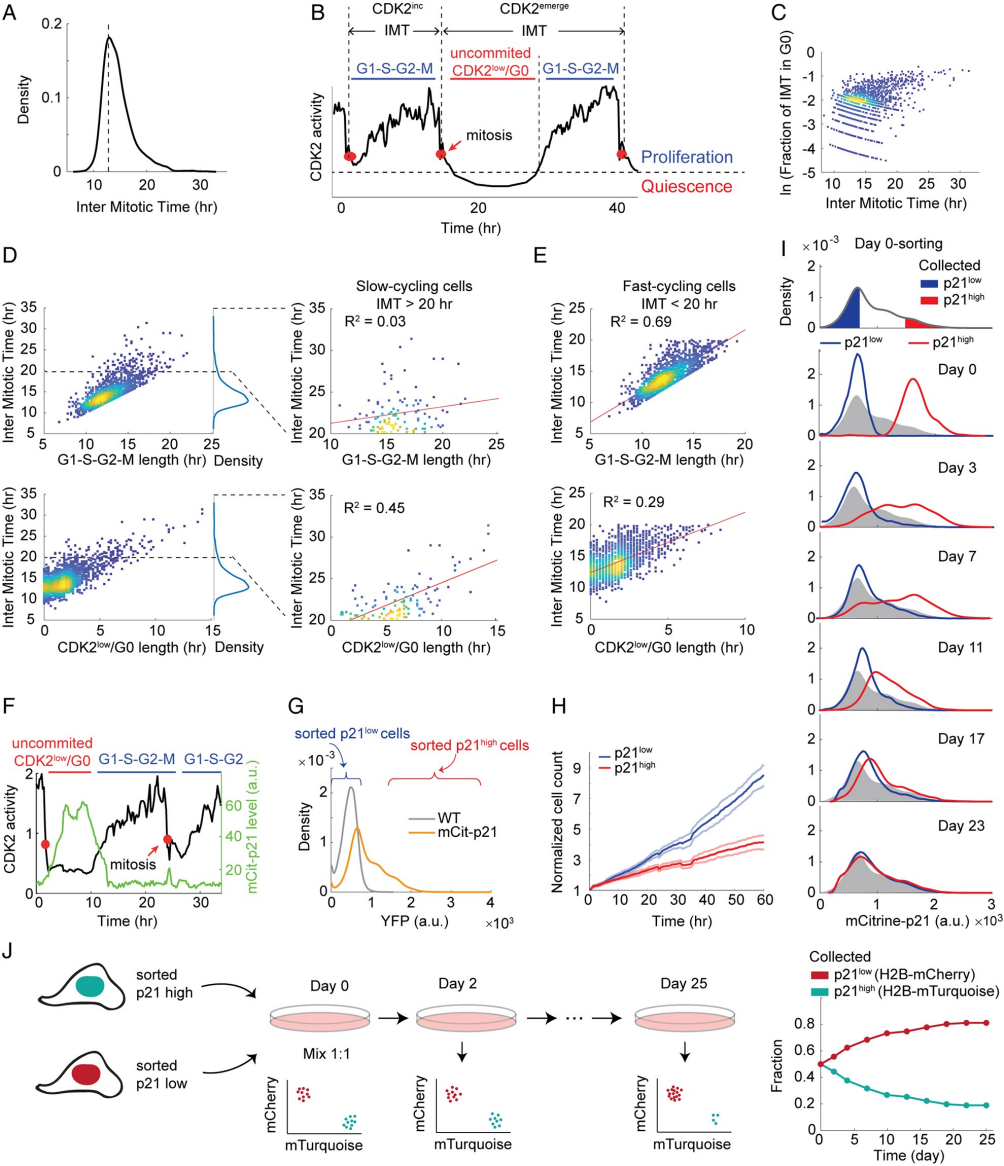
Isolation of a slow-cycling subpopulation. **(A)** Density plot of IMT in MCF10A cells. Density indicates relative number of cells displaying a particular IMT, with the area under the curve normalized to 1. Unperturbed MCF10A cells were imaged for 48 h to obtain IMTs for 2,000 cells. Mitosis was marked by chromosome segregation visualized with H2B-mTurquoise. Dotted line indicates the mode of the IMT distribution. (B) An example CDK2 trace used to display terminology. The first cell cycle is an example of a CDK2^inc^ cycle and the second cell cycle is a CDK2^emerge^ cycle, representing about 80% and 10%, respectively, of all cell cycles in the population. The remaining 10% of cells enter the CDK2^low^ state and remain there until the end of the imaging period (not shown). Red dots mark mitosis. (C) Density scatterplot of fraction of IMT spent in G0 versus IMT. The y-axis is in log scale. (D) Density scatterplot of IMT versus G1–S–G2–M length (top) and IMT versus G0 length (bottom). To the right of each plot, the data for slow-cycling cells are replotted from 20 to 35 h and fit by linear regression to determine the amount of variability in IMT explained by G0 duration versus G1–S–G2–M duration. G0 length explains 45% of the variation in IMT for slow-cycling cells. (E) Density scatterplot of IMT versus G1–S–G2–M length (top) and IMT versus G0 length (bottom) in fast-cycling cells (IMT < 20 h). (F) A representative single-cell trace of CDK2 activity and mCitrine-p21 in CDK2^low^/p21^high^ (first cell cycle) and CDK2^inc^/p21^low^ (second cell cycle) states. Red dots mark mitosis. (G) Density plot of mCitrine-p21 intensity in mCitrine-p21 knock-in cells. Density indicates relative number of cells displaying a particular YFP intensity with the area under the curve normalized to 1. Wild-type cells serve as a negative control. (H) Cell count (mean ± 95% confidence interval) of sorted p21^high^ and p21^low^ cells over 60 h imaging, starting from 30 h after sorting. Cell nuclei were computationally segmented based on H2B-mTurquoise images and counted using in-house Matlab scripts. *n* = 5 wells for each population. (I) p21^high^ and p21^low^ cells were sorted on Day 0 as indicated by the red and blue gates (top). The two subpopulations were then remeasured immediately after sorting (Day 0), or cultured and remeasured on Days 3, 7, 11, 17, and 23, with the distribution of p21 on the day of sorting reproduced in gray. Density indicates relative number of cells displaying a particular YFP intensity with the area under the curve normalized to 1. (J) Competition between sorted p21^high^ and p21^low^ cells. Cells were labelled with H2B-mTurquoise or H2B-mCherry and sorted into p21^high^ and p21^low^ populations, respectively. H2B-mTurquoise p21^high^ cells and H2B-mCherry p21^low^ cells were mixed at a ratio of 1:1 at Day 0. Population dynamics were monitored via the H2B labels for 25 d. Underlying data for this figure can be found in the BioStudies database under accession number S-BSST231. a.u., arbitrary unit; IMT, intermitotic time; YFP, yellow fluorescent protein.

To investigate the source of variation in cell-cycle length, we used an immortalized non-transformed human epithelial cell line, MCF10A, which has intact cell-cycle checkpoints. The intermitotic time (IMT) of individual MCF10A cells follows a distribution that peaks at 13 h with a long right tail (Fig 1A), confirming the existence of a slow-cycling subpopulation. We further confirmed that the slow-cycling subpopulation cannot be explained by experimental settings such as expressing tagged histones or phototoxicity from imaging (S1A–S1C Fig). The long IMT could either be due to entry into a long noncycling phase, such as G0, or due to a lengthened proliferative cell cycle consisting of G1–S–G2–M. To distinguish between these two possibilities, we used MCF10A cells expressing DHB-mVenus, a live-cell sensor of CDK2 activity, to mark the time at which CDK2 activity first begins to rise, a molecular event that we have previously shown to coincide with Rb hyperphosphorylation and cell-cycle commitment at the Restriction Point (R-point) (Fig 1B) [10].

As previously reported, the majority of cells increase their CDK2 activity shortly after anaphase and immediately commit to another cell cycle (Fig 1B, first cell cycle) [10]. As a consequence, the IMTs of this CDK2^inc^ subpopulation correlate well with time spent in G1–S–G2–M (Fig 1C–1E and S1A Fig). This CDK2^inc^ subpopulation also tends to have short IMTs and makes up the dominant mode in the IMT distribution in Fig 1A (Fig 1C). The cells in the right tail of the distribution, however, tend to spend long periods in the CDK2^low^ state (Fig 1C and 1D). Time spent in the CDK2^low^ state is the main contributor to the long IMT and explains 45% of the variation in IMT, in contrast to 3% explained by the length of G1–S–G2–M (Fig 1C and 1D and S1D Fig). Taken together, our data suggest that slow-cycling cells (those with long IMTs) result from prolonged transits through the CDK2^low^ state.

To isolate and profile the CDK2^low^ population, we took advantage of the previous finding that CDK2 activity strongly anticorrelates with p21 protein level (Fig 1F) [10,12,13], which enables us to separate the CDK2^low^ population by fluorescence-activated cell sorting (FACS) based on high p21 levels. We used an MCF10A cell line in which an mCitrine-p21 fusion protein is expressed from the endogenous p21 locus [12]. The distribution shape of mCitrine-p21 intensity resembles that of IMT with a long right tail: the majority of cells have low to zero levels of mCitrine-p21, similar to wild-type cells that do not express the mCitrine fusion protein, while a small subpopulation of cells have elevated p21 levels (Fig 1G). We used FACS to collect two subpopulations, p21^low^ and p21^high^, corresponding to CDK2^inc^ and CDK2^low^ cells, respectively (Fig 1G). We validated that the sorted p21^high^ subpopulation indeed expresses higher levels of p21 protein, and lower level of phospho-Rb (S1E Fig). We then followed the two sorted subpopulations by live-cell imaging for three days, starting from one day after the sorting, and confirmed that the p21^high^ subpopulation indeed proliferates more slowly than the p21^low^ subpopulation (Fig 1H), indicating a fitness disadvantage in optimal growth conditions.

How stable is the p21^high^ slow-cycling state? We previously showed that cells that pass through the CDK2^low^ state and then re-enter the cell cycle and divide are more likely to generate daughters that enter the CDK2^low^ state after mitosis [10]. We have also shown that daughter cells entering the CDK2^low^ state have mothers with longer IMTs [15]. Together, these results suggest that the slow-cycling state is heritable beyond a single generation.

To determine the longevity of the CDK2^low^/p21^high^ state, we sorted the p21^high^ and p21^low^ subpopulations and remeasured the distribution of p21 intensity of the two subpopulations every few days. Strikingly, the two subpopulations take about 3 wk to re-establish the steady-state p21 distribution (Fig 1I). To test whether both interconversion of the two subpopulations and a higher proliferation rate of p21^low^ cells contribute to re-establishing the steady-state p21 distribution, we labelled the mCitrine-p21 cell line with either H2B-mTurquoise or H2B-mCherry, and sorted them into p21^high^ and p21^low^ subpopulations, respectively (Fig 1J, left). The labelling of the two subpopulations allowed us to coculture them and monitor the population dynamics. If no outgrowth of the faster cycling p21^low^ cells occurs, the two colors should remain at the initial mixing fraction. However, we found that the fraction of H2B-mCherry, initially p21^low^ cells, gradually increased, consistent with fast-cycling p21^low^ cells outcompeting slow-cycling p21^high^ cells (Fig 1J, right). The fractions of two colors reached a steady state after 3 wk, a similar time frame to that of re-establishing the steady-state p21 distribution (Fig 1J, right). If no interconversion between CDK2^low^/p21^high^ and CDK2^inc^/p21^low^ cells occurs, we would expect that the H2B-mTurquoise, initially p21^high^ cells, will eventually be excluded from the population. In contrast to this scenario, at an initial mixing ratio of 1:1, the H2B-mTurquoise, initially p21^high^ cells, reached a steady-state fraction of 20% of the population (Fig 1J, right), suggesting that interconversion occurred. Moreover, this 20% of the population cannot be explained by a small fraction of p21^low^ cells in the collected p21^high^ subpopulation, given the sorting purity estimated by remeasuring the mCitrine-p21 intensity right after the initial sort (99.8% and 99.1% for p21^low^ and p21^high^ subpopulations, respectively). Therefore, convergence to the steady-state p21 distribution is partially driven by interconversion of p21^high^ cells and p21^low^ cells, and partially by outgrowth of faster-cycling p21^low^ cells over slower-cycling p21^high^ cells. These data suggest that although the p21^high^ state is reversible, it is a relatively long-lived state at the population level.

### Characterization of the transcriptome of spontaneous CDK2^low^ cells

We sought to characterize the CDK2^low^/p21^high^ slow-cycling state by performing RNA sequencing (RNA-seq) on the sorted subpopulations described above. To minimize the bias from cell-cycle phase differences, we sorted only G0/G1 cells using the geminin sensor of the fluorescence ubiquitination cell cycle indicator (FUCCI) [18]. The geminin sensor is absent in G0/G1 cells and accumulates from S phase to mitosis [18]. We confirmed that the intensity of the geminin sensor shows a bimodal distribution, and that the geminin-negative cells almost exclusively have 2N DNA content (S1F and S1G Fig). We collected only the geminin-negative cells, and sorted these into p21^high^ and p21^low^ subpopulations (S1G Fig). To reduce clonal effects, we carried out the sorting followed by RNA-seq in two different clones of mCitrine-p21 cells, one clone with one wild-type *CDKN1A* allele and one mCitrine knock-in allele (clone 2e2), and the other clone with one mCitrine knock-in allele and one knock-out allele (clone 3b6) [12]. Despite the copy number difference of the gene, the two clones express similar levels of p21 at the single-cell level (S1H Fig).

The genes that are up-regulated in the CDK2^low^/p21^high^ population are most highly enriched in transcriptional targets of p53 (Table 1, S1 and S2 Tables), consistent with the fact that p53 is a transcription factor for p21 [19,20]. The up-regulation of p53 target genes (and p53 protein itself, S2A and S2B Fig) is also consistent with the observation that about half of the CDK2^low^ subpopulation has 53BP1 nuclear bodies and γH2AX foci, markers of DNA lesions (S2C Fig) [15,16].

**Table 1:** Pathways up-regulated in spontaneously quiescent CDK2^low^/p21^high^ cells relative to G1-phase CDK2^inc^/p21^low^ cells.

Pathway ID	Pathway Name	Adjusted *p*-value
hsa04115	p53 Signaling pathway	4.10 × 10^−10^
hsa04137	Mitophagy	0.000168
hsa04068	FoxO signaling pathway	0.000238
hsa04510	Focal adhesion	0.010779
hsa04140	Autophagy	0.015879
hsa00600	Sphingolipid metabolism	0.020937
hsa04512	ECM-receptor interaction	0.020937
hsa04142	Lysosome	0.022506
hsa04360	Axon guidance	0.033331

Abbreviations: ECM, extracellular matrix; ID, identifier.

Additionally, the mitophagy and autophagy-lysosome pathways are up-regulated in the CDK2^low^/p21^high^ cells (Table 1), a result we validated with the observation of up-regulated PINK1 and LAMP1 proteins by western blot (S2A and S2B Fig). This result is consistent with previous findings that these pathways protect quiescent cells from oxidative stress and mitochondrial dysfunction, and have been suggested to be important for the maintenance of quiescence [14,21].

We also observed that CDK2^low^/p21^high^ cells show increased expression of extracellular matrix (ECM) receptors, such as E-cadherin and integrin, and ECM components such as collagen, fibronectin, and laminin (Table 1, S1 and S2 Tables). This observation suggests that CDK2^low^/p21^high^ cells remodel and interact with their extracellular environment more extensively than CDK2^inc^/p21^low^ cells. Similarly, many receptors on the plasma membrane and secreted signaling ligands are up-regulated in CDK2^low^/p21^high^ cells, including the NOTCH family, WNT5A, TGF-β receptors, and BMP family (Table 1, S1 Table), suggesting increased cell–cell communication in these cells.

Interestingly, a few markers of cancer and normal tissue stem cells are up-regulated in the CDK2^low^/p21^high^ cells (S1 Table), which often function to protect stem cells from environmental insults. Examples include elevated levels of aldehyde dehydrogenase ADLH1A3 to detoxify drugs [22,23]; increased expression of FOXO1, a transcription factor implicated in the regulation of an anti-reactive oxygen species gene expression program [24]; and up-regulation of ATP-binding cassette (ABC) transporters that are capable of drug efflux [25]. These shared molecular features between slow-cycling cells and cancer stem cells suggest that slow cycling may be an intrinsic property of cancer stem cells that protects them from drug treatment.

The pathways that are most significantly down-regulated in CDK2^low^/p21^high^ cells relative to CDK2^inc^/p21^low^ cells are DNA replication and cell cycle (for example, the minichromosome maintenance protein complex [MCM] complex and the DNA replisome complex) (Table 2, S1 and S2 Tables). Although both sorted subpopulations were geminin negative and therefore in G0/G1 at the time of sorting and sequencing, the data suggest that the p21^low^ subpopulation was preparing for DNA replication and cell-cycle progression, whereas the p21^high^ subpopulation appears less ready to complete the cell cycle. Consistent with our previous finding that the CDK2^low^/p21^high^ cells are not senescent [13,15], the cellular senescence pathway is down-regulated in these cells.

**Table 2:** Pathways down-regulated in spontaneously quiescent CDK2^low^/p21^high^ cells relative to G1-phase CDK2^inc^/p21^low^ cells.

Pathway ID	Pathway Name	Adjusted *p*-value
hsa03030	DNA replication	1.61 × 10^−21^
hsa04110	Cell cycle	4.02 × 10^−21^
hsa03013	RNA transport	1.41 × 10^−17^
hsa03040	Spliceosome	1.76 × 10^−16^
hsa03430	Mismatch repair	5.04 × 10^−9^
hsa03420	Nucleotide excision repair	3.05 × 10^−6^
hsa03410	Base excision repair	1.05 × 10^−5^
hsa00240	Pyrimidine metabolism	3.07 × 10^−5^
hsa03440	Homologous recombination	0.000684
hsa04218	Cellular senescence	0.001197
hsa04114	Oocyte meiosis	0.001613
hsa03008	Ribosome biogenesis in eukaryotes	0.001703
hsa00230	Purine metabolism	0.022246

Abbreviations: ID, identifier.

Four major DNA repair pathways (mismatch repair, nucleotide excision repair, base excision repair, and homologous recombination) are down-regulated in CDK2^low^/p21^high^ cells, an initially surprising finding given that these cells have been shown to have increased levels of DNA damage markers relative to CDK2^inc^/p21^low^ cells (S2C Fig) [15,16]. A closer inspection of the gene lists reveals that the down-regulated genes mostly function not in DNA damage detection (e.g., p53 target genes) but in actual repair of DNA damage, and often require S-phase entry for their expression (e.g., DNA polymerases and ligases) (S1 Table) [26]. The down-regulation of these pathways in CDK2^low^/p21^high^ cells therefore likely results from a lack of preparation for S phase in CDK2^low^/p21^high^ cells relative to CDK2^inc^/p21^low^ cells.

### Analysis of five triggers of quiescence reveals five distinct quiescent states

To compare the transcriptome of the spontaneously quiescent CDK2^low^ cells with more canonical quiescent states, we also performed RNA-seq on cells forced into quiescence for 48 h using traditional (serum starvation, contact inhibition) [14] and somewhat less traditional methods (Mek inhibition, CDK4/6 inhibition) (S3A Fig). We confirmed that these perturbations forced cells out of the cycling state, marked by reduced mRNA levels of proliferation markers, such as Ki67 and Cyclin B1 (S1 Table), and absence of phospho-Rb and 5-ethynyl deoxyuridine (EdU) incorporation (a marker for DNA synthesis) (S3B Fig). We note that under contact inhibition and CDK4/6 inhibition, a small fraction of cells are phospho-Rb^high^ and EdU^+^, likely explaining the lack of significance of some cell-cycle gene down-regulation, especially genes that are lowly expressed in MCF10A cells, such as CCNE1 and E2F1. We also verified that these perturbations did not force cells into senescence, because the cells can readily revert back to proliferation within 24 h of restoring full-growth conditions, with a similar fraction of phospho-Rb^high^ and EdU^+^ as control cells (S3B Fig).

Because these quiescent states are induced by different pathways, we reasoned that genes that are differentially regulated in these various forms of quiescence will likely reveal the causes of quiescence, whilst genes that are commonly regulated in all forms of quiescence may indicate consequences of quiescence. For example, we recently showed that the bifurcation of Ki67 levels follows, and therefore is a consequence of, the proliferation-quiescence decision [11], which is consistent with Ki67 being down-regulated in all five forms of quiescence examined here (S1 Table).

**Fig 2:**
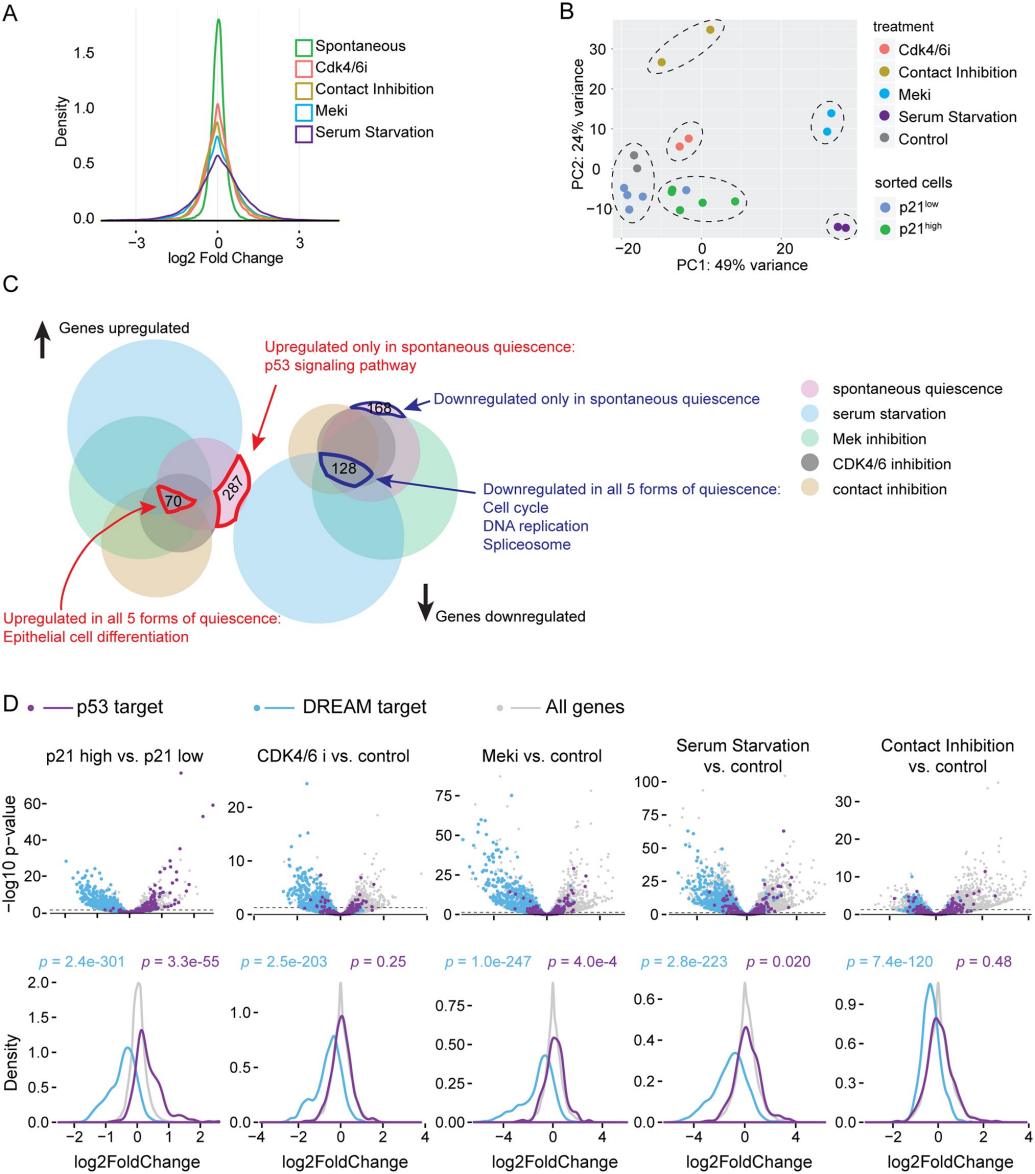
Transcriptome of spontaneous quiescence versus forced quiescence. (A) Density plot of log2 fold change of transcriptomes in various forms of quiescence. For spontaneous quiescence, the fold change is calculated as the ratio of expression levels in sorted p21^high^ versus sorted p21^low^ cells. For other forms of quiescence, the fold change is perturbed cells versus control cells. (B) PCA analysis of all samples, for mCitrine-p21 knock-in clone 2e2. Control samples are untreated, unsorted cells. Dashed ovals indicate clusters. (C) Venn diagram of gene sets differentially regulated in five forms of quiescence. Note: the area of each set does not strictly correlate with the number of genes contained within. (D) Volcano plot (top) and distribution of log2 fold change (bottom) of p53 targets (purple) and DREAM complex targets (blue). Dash lines mark *p* = 0.05 threshold for gene differential regulation (top). The *p*-values from Mann–Whitney *U* test indicate significant down-regulation of DREAM targets in all forms of quiescence and significant and strong up-regulation of p53 targets in spontaneous quiescence only. Underlying data for this figure can be found in the GEO database under accession number GSE122927. DREAM, dimerization partner, RB-like, E2F4, and multi-vulval class B; GEO, Gene Expression Omnibus; PCA, principal component analysis.

The distribution of expression fold-change immediately shows that different quiescence-induction methods perturb cells to dramatically different degrees, with serum starvation causing the widest range of differential expression, followed by Mek inhibition, contact inhibition, and CDK4/6 inhibition, with spontaneous quiescence showing the least differential expression relative to cycling cells (Fig 2A). This is consistent with the fact that at one extreme, serum starvation will lead to loss of mitogenic signaling from many receptor signaling pathways, whereas CDK4/6 inhibition is a highly specific perturbation, and spontaneously quiescent CDK2^low^ cells are not even artificially perturbed.

Principal component analysis (PCA) of transcriptome data from the spontaneously quiescent CDK2^low^ state and the four forced-quiescence states revealed five distinct clusters that correlated with the quiescence-induction method (Fig 2B), although we note there is clonal variation in the positioning of the clusters (S4A Fig). The PCA analysis therefore suggests that the five different triggers of quiescence lead to five transcriptomically distinct states of quiescence, at least at the 48 h time point examined here. Consistent with a previous study [14], these data suggest that quiescence is not a single state. The first principle component separates serum starvation and Mek inhibition from other samples (Fig 2B and S4A Fig), indicating that the dominant feature separating the five types of quiescence is the mitogen-activated protein kinase (MAPK) pathway, which accounts for 35%–49% of the variance (Fig 2B and S4A Fig). We also note that p21^high^ and p21^low^ cells are not far separated in the PCA, consistent with their relatively small transcriptional differences compared with that between control and forced-quiescence samples (Fig 2A and 2B).

### Down-regulation of DREAM targets is a consequence of quiescence

Seventy genes are transcriptionally up-regulated in all five forms of quiescence (Fig 2C, S4B Fig and S3 Table). However, no particular signaling pathway is enriched in this group of genes, and the only enriched biological process in Gene Ontology (GO) is “epithelial cell differentiation”. Given that MCF10A cells originate from epithelium, this result suggests that genes universally turned on in all quiescence conditions may be constrained by the cell identity. Consistent with this interpretation, this list of genes overlaps poorly with a previously published “fibroblast quiescence program”, which includes 116 genes consistently up-regulated in three quiescence conditions in fibroblast cells [14], with only one overlap between the two lists (S4 Table).

Among the 128 genes that are universally downregulated in all forms of quiescence, the enriched pathways are DNA Replication, Spliceosome and Cell Cycle, part of the “fibroblast quiescence program” in a previous study (Fig 2C, S4B Fig and S3 Table) [14]. Highly enriched in these genes are targets of the dimerization partner, RB-like, E2F4, and multi-vulval class B (DREAM) complex, which includes transcriptional targets of E2F1, 2, 3 in the early cell cycle and targets of the MuvB complex with B-Myb or FOXM1 (MMB-FOXM1) in the late cell cycle [27] (S5 Table). In quiescence, the DREAM complex binds to the promoter of its targets and suppresses their expression. Once cells commit to the cell cycle, the DREAM complex disassembles and liberates the promoter for E2F- and, later, MMB-dependent transcription [28]. Notably, expression of DREAM targets is repressed in all forms of quiescence examined here (Fig 2D), suggesting that a DREAM-dependent suppression program is active and that it is likely a consequence of quiescence.

**Fig 3:**
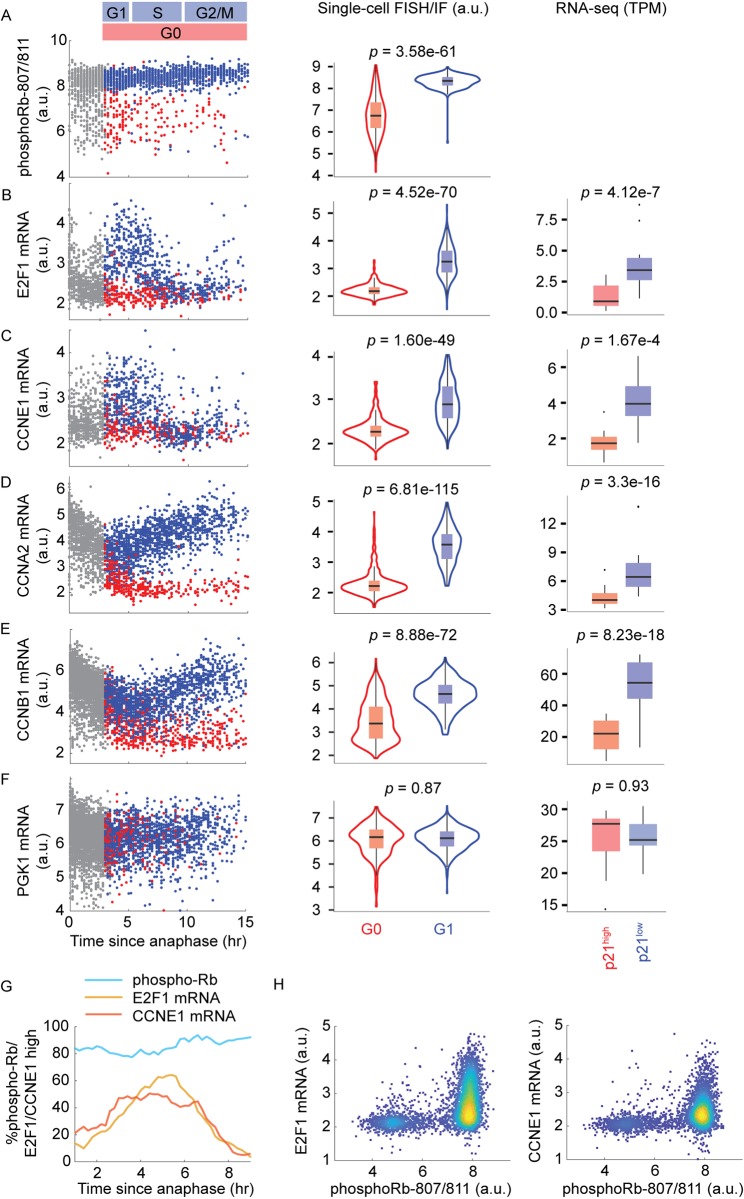
mRNA dynamics of DREAM targets in proliferating and spontaneously quiescent MCF10A cells. (A-F) Column 1: time-lapse imaging of CDK2 activity in asynchronous cells was followed by fixation and IF or FISH staining for the indicated protein or mRNA. Protein or mRNA signals were then reconstructed as a function of time since anaphase for CDK2^inc^ cells (blue dots) and CDK2^low^ cells (red dots). Gray dots indicate cells too early in the cell cycle to be classified as CDK2^inc^ or CDK2^low^. The y-axes are in log scale. Column 2: distribution of mRNA or protein levels in G1 (CDK2^inc^ cells within 3–5 h after mitosis) versus G0 (all CDK2^low^ cells). The *p*-values from Mann–Whitney *U* test indicate significant difference of Rb phospho-807/811, E2F1 mRNA, CCNE1 mRNA, CCNA2 mRNA, and CCNB1 mRNA levels in G0 versus G1 cells. The y-axes are in log scale. Column 3: box plot of mRNA level in p21^high^ versus p21^low^ cells measured by RNA-seq; data from both mCitrine-p21 clones are pooled to generate *n* = 10 samples for each condition. The *p*-values from DEseq2 indicate significant difference of E2F1 mRNA, CCNE1 mRNA, CCNA2 mRNA, and CCNB1 mRNA in p21^high^ versus p21^low^ cells (see also S1 Table). (G) Percentage of E2F1^high^, CCNE1^high^, or phospho-Rb^high^ cells as a function of time since anaphase, using thresholds determined by the Otsu method [29]. (H) Density scatterplots of mRNA levels of E2F1 or CCNE1 versus phospho-Rb S 807/811 in cells with 2N DNA content. Both axes are in log scale. Underlying data for this figure can be found in the BioStudies database under accession number S-BSST231. a.u., arbitrary unit; DREAM, dimerization partner, RB-like, E2F4, and multi-vulval class B; RNA FISH, RNA fluorescence in situ hybridization; IF, immunofluorescence; RNA-seq, RNA sequencing; TPM, transcripts per million.

To test this notion experimentally, we considered that a logical requirement of differential gene expression being a consequence of quiescence is that the differential expression occurs after the cell’s decision to enter quiescence. We therefore sought to validate our RNA-seq results using single-cell time-lapse microscopy, followed by RNA fluorescence in situ hybridization (RNA FISH) or immunofluorescence (IF), as in Gookin and colleagues [13]. By tracking hundreds of asynchronously cycling cells, we can populate the trajectory of the mRNA or protein of interest and reproduce the dynamics of that gene’s expression in single cells throughout the cell cycle [10,13]. We considered four DREAM targets, E2F1, CCNE1, CCNA2, and CCNB1, as well as PGK1 as a negative control, because it is not a DREAM target [27] (Fig 3 and S5 Fig).

To determine the relative timing of the proliferation-quiescence decision and the bifurcation of DREAM-target mRNAs, we first confirmed previous reports describing two subpopulations of cells with distinct levels of phosphorylated Rb that are visible immediately after anaphase [10,12,13] (Fig 3A). The majority of cells (about 80%) have high levels of phospho-Rb, corresponding to the fraction that will become CDK2^inc^ cells (Fig 3G). A smaller subset of cells (about 20%) have low levels of phospho-Rb, corresponding to the fraction that will become CDK2^low^ cells. These two populations are already discernible in late anaphase (Fig 3A) [12]. By contrast, the mRNA levels of E2F targets, E2F1 and CCNE1, are low in the vast majority of cells at anaphase (Fig 3B, 3C and 3G). From there, they begin to rise in CDK2^inc^ cells, peaking at about 5 h after anaphase, whereas they remain low in CDK2^low^ cells (Fig 3B and 3C). Thus, CDK2^inc^ cells have already committed to proliferation at anaphase (as marked by high levels of phosphorylated Rb), but only up-regulate E2F1 and CCNE1 a few hours later. To further demonstrate this, we stained for phospho-Rb and E2F1 or CCNE1 mRNA in the same cells and identify a subpopulation of phospho-Rb^high^/E2F1^low^/CCNE1^low^cells, indicating that Rb hyperphosphorylation occurs prior to E2F and CCNE1 transcription (Fig 3H). mRNA levels of CCNA2 and CCNB1, DREAM targets that function later in the cell cycle, diverge even later in the CDK2^inc^ versus CDK2^low^ subpopulations, at 5–7 h after anaphase (Fig 3D and 3E). Together, these results confirm that differential expression of DREAM targets between the CDK2^inc^ and CDK2^low^ subpopulations occurs after the proliferation-quiescence decision and therefore is a consequence of the proliferation-quiescence decision.

### Stress responses cause entry into the spontaneous CDK2^low^ state

We next examined the RNA-seq data to find causes of spontaneous entry into the CDK2^low^ state from genes uniquely up- or down-regulated in cells in this state. There are 287 genes up-regulated and 168 genes down-regulated in the spontaneous CDK2^low^ cells that are not differentially expressed relative to other forms of quiescence (Fig 2C and S4B Fig). The only enriched pathway within this set of genes is the p53 signaling pathway (Fig 2C and S4B Fig), suggesting that up-regulation of p53 signaling is a cause of spontaneous quiescence entry.

**Fig 4:**
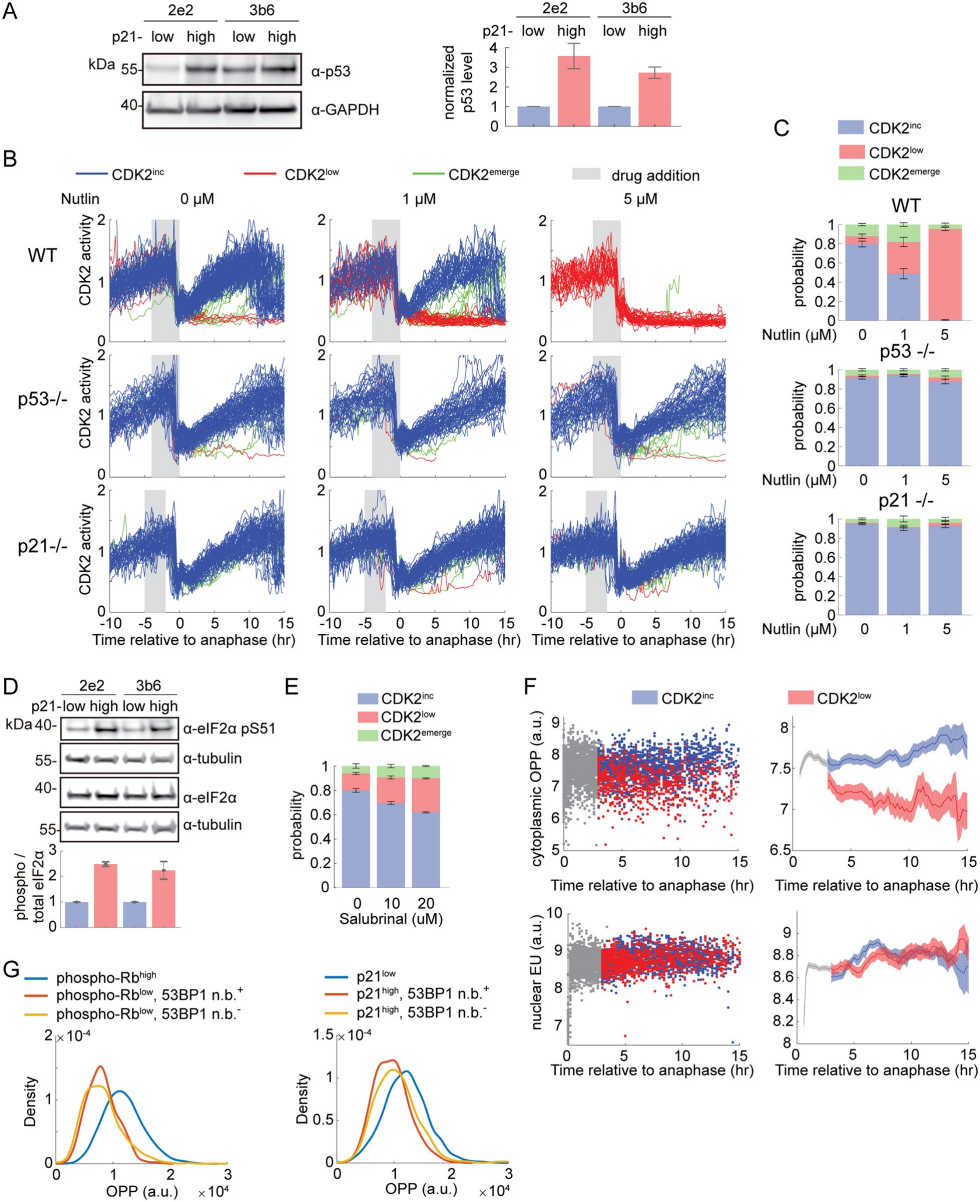
Entry into spontaneous CDK2^low^ quiescence is caused by stress responses. (A) Western blot of sorted p21^high^ and p21^low^ cells shows that p53 protein levels are increased in p21^high^ cells in both mCitrine-p21 clones. The quantification is duplicated from S2A Fig. (B) Single-cell traces from WT, p53-null (*p53*^*−/−*^), or p21-null (*p21*^*−/−*^) MCF10A cells under control condition or treated with 1 μM or 5 μM Nutlin. Traces are computationally aligned to the time of anaphase, and only cells receiving drug in G2 phase are plotted. Traces are colored based on the fate of daughter cells: CDK2^inc^ in blue, CDK2^low^ in red, and CDK2^emerge^ in green. See Materials and methods for classification criteria. A total of 300 traces are plotted in each condition. (C) Percentage of CDK2^inc^, CDK2^low^, and CDK2^emerge^ cells in conditions in (B). Data are mean ± SEM across 8 wells. (D) Western blot of sorted p21^high^ and p21^low^ cells shows that phosphorylation of eIF2α at S51 is increased in p21^high^ cells. Quantification of the blot is shown below, with phospho-eIF2α and total eIF2α normalized by tubulin levels. (E) Percentage of CDK2^inc^, CDK2^low^, and CDK2^emerge^ cells in conditions in which mother cells were treated with 0, 10, or 20 μM salubrinal 5–15 h prior to mitosis. Data are mean ± SEM across 4 wells for each condition. (F) Time-lapse imaging of CDK2 activity in asynchronous cells was followed by an OPP or EU pulse of 24 min and fixation. Incorporated OPP or EU was quantified by Click-iT assay and fluorescence microscopy. The incorporated OPP (top) or EU (bottom) concentration was then reconstructed as a function of time since anaphase. Left, single cell data: CDK2^inc^ cells (blue dots), CDK2^low^ cells (red dots), cells too early in the cell cycle to classify as CDK2^inc^ or CDK2^low^ (gray dots). Right, moving average (mean ± 95% confidence interval). The y-axes are in log scale. (G) Density distribution of OPP in three subpopulations of cells: phospho-Rb^high^/p21^low^; phospho-Rb^low^/p21^high^ /53BP1 n.b.^+^; and phospho-Rb^low^/p21^high^/53BP1 n.b.^−^. Underlying data for this figure can be found in the BioStudies database under accession number S-BSST231. a.u., arbitrary unit; EU, 5-ethynyl uridine; n.b., nuclear body; OPP, O-propargyl-puromycin; SEM, standard error of the mean; WT, wild-type.

We first confirmed that p53 protein levels are higher in p21^high^/CDK2^low^ cells versus p21^low^/CDK2^inc^ cells and that p53 transcriptional targets are strongly induced in p21^high^ cells but not in other forms of quiescence (Figs 2D and 4A, S2A and S2B Fig). As previously reported, knockout of p53 or p21 eliminates the spontaneous CDK2^low^ subpopulation (Fig 4B and 4C) [10,15–17]. Conversely, increasing p53 protein levels in G2 by adding Nutlin (an inhibitor of MDM2, a ubiquitin ligase responsible for p53 degradation) can force wild-type cells into a CDK2^low^ state after mitosis (Fig 4B and 4C) [16,17]. Therefore, activation of p53 is necessary and sufficient for entering the spontaneous CDK2^low^ state.

Activation of the p53 pathway is consistent with the finding that CDK2^low^ cells harbor increased DNA damage relative to CDK2^inc^ cells [15,16]. However, only approximately half of the cells in the CDK2^low^ state show signs of DNA damage [15,16], raising the question of whether other stresses may be involved in promoting the remainder of the transits through the CDK2^low^ state. We reasoned that the specific stress may vary from cell to cell, but that stress signaling can converge at stress hubs such as p53 and emerge as a subpopulation phenotype. We therefore examined a second stress-response pathway that integrates multiple stresses, the ISR-eIF2α pathway. Four upstream kinases, GCN2, PERK, HRI, and PKR, which sense amino acid deprivation, endoplasmic reticulum (ER) stress, heme deprivation, and viral infection, respectively, phosphorylate eIF2α, a critical component of translation initiation. Phosphorylated eIF2α impairs general cap-dependent translation, but enhances translation of specific downstream effectors of the ISR, such as ATF4, a transcription factor for a group of stress-responsive genes [30].

We first attempted to examine the overall expression level of ATF4 target genes in our dataset. However, unlike p53, for which chromatin immunoprecipitation sequencing (ChIP-Seq) and global run-on sequencing (GRO-Seq) studies have systematically discovered direct transcriptional targets [27,31], no such dataset is available for ATF4. We therefore constructed from the literature a list of six ATF4 transcriptional targets that are expressed in MCF10A cells [32,33]. We found that five of these six genes are up-regulated in CDK2^low^/p21^high^ cells, whereas they are regulated in a less consistent way in other forms of quiescence, suggesting activation of ATF4 in CDK2^low^/p21^high^ cells but not in other forms of quiescence (S6A Fig). ATF4 protein was below the detection limit of our antibody in unperturbed conditions (S6B Fig). We therefore examined phosphorylation of eIF2α, the upstream inducer of ATF4. Consistent with the RNA-seq result, we detected much stronger phosphorylation of eIF2α in CDK2^low^/p21^high^ cells relative to CDK2^inc^/p21^low^ cells (Fig 4D). Taken together, these data indicate that the ISR-eIF2α pathway is activated in CDK2^low^ cells.

We next examined whether inhibition of eIF2α dephosphorylation would lead to an increase in the fraction of cells entering the CDK2^low^ state. We imaged cells expressing the CDK2 activity sensor in unperturbed conditions for 16 h, followed by treatment with salubrinal, an inhibitor of the eIF2α phosphatase Gadd34/PP1 [34], and further imaging for another 24 h. Treatment with salubrinal caused an increased fraction of cells to enter into a CDK2^low^ state after mitosis (Fig 4E), suggesting that activation of the ISR-eIF2α pathway can force cells into a CDK2^low^ quiescence.

What is the relationship between ISR-activated and DNA-damaged CDK2^low^/p21^high^ cells? Can ISR activation account for the cells transiting through the CDK2^low^ state that do not show signs of DNA damage? Answering these questions requires a single-cell readout of the ISR; however, the ATF4 and phospho-eIF2α antibody signals are not detectable by IF in unperturbed cells. Instead, we measured the global translation rate in single cells using the O-propargyl-puromycin (OPP) assay, in which an alkyne analog of puromycin, OPP, is incorporated into nascent peptides and can later be visualized by fluorescence microscopy using a copper-catalyzed alkyne-azide cycloaddition (CuAAC) reaction with a fluorescent azide [35]. To compare OPP incorporation between CDK2^low^ and CDK2^inc^ cells, we imaged cells for 24 h by time-lapse microscopy to identify each cell’s CDK2 activity state, pulsed cells for 24 min with OPP, and immediately fixed the cells with paraformaldehyde. We then matched each fixed cell back to its live-cell trace, thereby linking its CDK2 activity state to its rate of protein translation. We found that CDK2^low^ cells have a significantly lower translation rate than CDK2^inc^ cells (Fig 4F, top), consistent with their activation of the ISR. In contrast to translation rate, we detect no difference in transcription rate between CDK2^low^ cells and CDK2^inc^ cells (Fig 4F, bottom, and S6C Fig), using a 5-ethynyl uridine (EU) incorporation assay similar to the OPP incorporation assay [36]. This suggests that the reduced translation rate in CDK2^low^ cells is likely a specific signaling event, consistent with activation of the ISR in these cells.

We therefore used OPP incorporation as single-cell readout of the ISR to assess overlap in the activation of DNA damage and ISR stress pathways. We co-stained cells with proliferation/quiescence markers phospho-Rb/p21, DNA damage marker 53BP1, and ISR marker OPP. Consistent with our observation in CDK2^low^ cells (Fig 4F, top), phospho-Rb^low^/p21^high^ cells show reduced levels of OPP incorporation compared with phospho-Rb^high^/p21^low^ cells. This reduction of translation is apparent in phospho-Rb^low^/p21^high^ subpopulations with and without 53BP1 nuclear bodies (Fig 4G), indicating that activation of the ISR is independent of DNA damage. Therefore, activation of the ISR could account for some of the cells entering the CDK2^low^ state without DNA damage. However, we have not yet found a way to eliminate the ISR-associated slow-cycling state (including via small interfering RNA [siRNA] knockdown of the four eIF2α kinases, S6D and S6E Fig), which would be necessary to show that endogenous activation of the ISR indeed causes entry into the CDK2^low^ state.

### The ability to enter spontaneous quiescence fortifies cells against future stress

**Fig 5:**
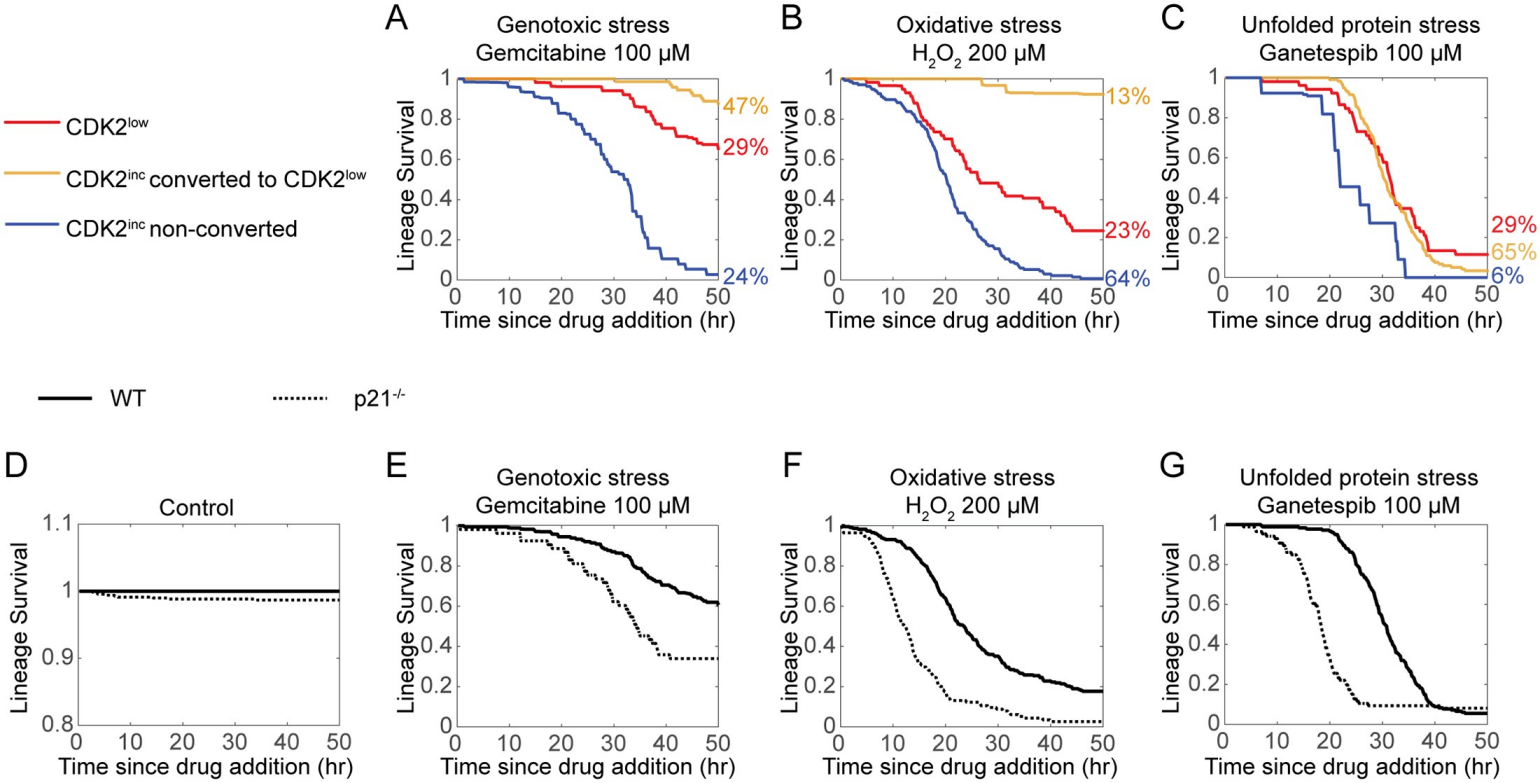
Entry into the CDK2^low^ state protects against exogenous stress. MCF10A cells were imaged in unperturbed conditions (D) or challenged with genotoxic stress (A, E), oxidative stress (B, F), or unfolded protein stress (C, G). Lineage survival is plotted as a Kaplan–Meyer curve (*n* = 20–200 cells per condition). (A-C) Lineage survival of cells that are CDK2^low^ at the time of drug addition (red), cells that are CDK2^inc^ at the time of drug addition but convert to CDK2^low^ later (either by division into CDK2^low^ daughters after mitosis, or because CDK2 activity dropped below 0.6 without a mitosis, yellow), or cells that are CDK2^inc^ until their death or the end of the imaging period (blue). The percentage of each cell category is indicated to the right. (D-G) Lineage survival of wild type versus *p21*^*−/−*^ cells. Underlying data for this figure can be found in the BioStudies database under accession number S-BSST231. WT, wild-type.

The essence of a stress response is to protect cells from stress. We hypothesized that cells in the spontaneous CDK2^low^ state would be more resistant to stress than their proliferating CDK2^inc^ counterparts. We initially sought to sort p21^high^/CDK2^low^ cells from p21^low^/CDK2^inc^ cells and test their resistance to exogenous stresses. However, applying exogenous stress rapidly converts many (17%–92%, depending on the stress) CDK2^inc^ cells into CDK2^low^ cells that may activate the same stress response as the spontaneous CDK2^low^ cells and hence obscure the result. We therefore used live-cell imaging to follow the fate of cells after applying exogenous stresses and computationally sorted the population into three categories: (1) cells that are CDK2^low^ at the time of stress addition; (2) those that are CDK2^inc^ at the time of stress addition but convert to CDK2^low^ in response to the stress, either by dividing into CDK2^low^ daughters or by dropping their CDK2 activity without a mitosis; and (3) those that are CDK2^inc^ at the time of the stress and remain CDK2^inc^ until their death or the end of the imaging period. Remarkably, in all three stress conditions, genotoxic stress, oxidative stress, or unfolded protein stress, the cells that persist in the CDK2^inc^ state have significantly worse survival than cells existing in or converting to the CDK2^low^ state (Fig 5A–5C), suggesting that the CDK2^low^ state indeed enables cell survival under stressful conditions. Thus, while CDK2^low^ cells have a fitness disadvantage under optimal growth conditions (Fig 1H), they have a fitness advantage under stressful conditions (Fig 5A–5C).

We further hypothesized that if entering quiescence is an essential part of the stress response, loss of the ability to enter quiescence should render cells particularly susceptible to exogenous stresses. We tested this idea using *p21*^*−/−*^ MCF10A cells, which are not able to enter the spontaneous CDK2^low^ state but should remain capable of activating other stress response pathways [10]. In unperturbed conditions, cell death occasionally occurred at a rate of 1.5% ± 0.2% per 50 h in *p21*^*−/−*^ cells, but never occurred in wild-type cells during the 50-h imaging period (Fig 5D, S1 and S2 Movies). Upon challenge by genotoxic stress, oxidative stress, or unfolded protein stress, *p21*^*−/−*^ cells displayed substantially worse survival than wild-type cells (Fig 5E–5G, S3 and S4 Movies). This result indicates that *p21*^*−/−*^ cells are less tolerant to a wide range of stresses and that the ability to enter a quiescent CDK2^low^ state in response to stress represents a survival advantage in stressful conditions.

## Discussion

Slow-cycling subpopulations have been implicated in antibiotic resistance in bacteria and drug resistance in cancer [3–5]. In bacteria, slow cycling has been proposed as a bet-hedging strategy, in which phenotypic variation improves long-term fitness by increasing survival through unpredictable environments, a beneficial trait that would have been selected by evolution. In the case of bet hedging, slow or fast cycling is a stochastic decision for a cell. In this work, we have investigated the origins of a slow-cycling subpopulation in a noncancerous human cell line and found that it largely results from a prolonged transit through a CDK2^low^ quiescent state. We further show that the CDK2^low^ state originates from activation of stress-response pathways, a deterministic molecular cause.

In addition to DNA damage-induced up-regulation of p21, p21^high^ cells also show activation of the ISR. Although it has been reported that p21 can be activated by the ISR via transcription- and translation-based mechanisms under exogenous stresses [37–39], precise determination of the connection between p21 and the ISR under spontaneous/endogenous stress conditions will require development of more sensitive detection methods for ATF4 and phospho-eIF2α in unperturbed single cells. While ISR activation appears to be independent of DNA damage as marked by 53BP1 nuclear bodies, it is not independent of p53, because p53 is required for entry into the spontaneous CDK2^low^ state (Fig 4B and 4C) [16,17]. The discrepancy between 53BP1 nuclear bodies (DNA damage) and p53 implies that p53 senses stresses beyond DNA damage. This is supported by the findings that p53 can be activated by stresses independent of DNA damage, such as nucleolar stress, hypoxia, and oncogene activation [40]. Thus, our current results implicate p53 and p21 as downstream effectors of stress signaling beyond their canonical role in response to DNA damage.

While genetic knockout of p53 or p21 eliminates this CDK2^low^ subpopulation, we have not found a way to reduce entry into the spontaneous CDK2^low^ state by reducing potential stresses. Therefore, it remains unproven whether cellular stress explains all transits through the spontaneous CDK2^low^ state. It is possible that each type of stress only accounts for a small fraction of cells entering the spontaneous CDK2^low^ state. Alternatively, cell stress may be intrinsically associated with the stochastic nature of biochemical reactions in cells (e.g., DNA replication, transcription, and metabolism) [41,42] and therefore may be impossible to eliminate. A third interpretation is that spontaneous CDK2^low^ cells hijack stress-response pathways to enter a noncycling state in the absence of stress. Both the p53 pathway and the eIF2α pathway have been implicated in maintaining stem cell quiescence in mice as a physiological function [43,44]. In this case, cells may simply activate these pathways without stress in response to extrinsic cues, or in a stochastic manner, as a means to trigger quiescence.

The bet-hedging theory relies on the fact that slow-cycling micro-organisms are more resistant to harsh environments [1]. In the case of cancer cells, an intuitive interpretation is that dormant cancer cells escape treatment by avoiding S phase, which is often the target of chemotherapy and radiotherapy [45]. Our findings show that the ability to enter spontaneous quiescence is beneficial not only in genotoxic stress conditions but also in diverse other stress conditions, suggesting that quiescence actively protects cells from exogenous stress. Together with our finding that activation of stress responses causes entry into spontaneous quiescence, these results indicate that the nature of spontaneous quiescence is a stress response to various endogenous stresses, given that these cells are in optimal growth conditions without exogenously added stress. Our observation that blocking entry into spontaneous quiescence (by p21 knockout) compromises the protective function of the stress response suggests that the slow-cycling state is not a passive consequence of stress, but an essential and inseparable part of the stress response that fortifies cells against various stresses.

The stress response is perhaps one of the most conserved biological functions across all kingdoms of life. Our data suggest that reduced proliferation is hardwired into the stress response in human cells. With similar findings in bacteria and yeast [1,8], we propose that this hardwiring of a slow-cycling state is as conserved as the stress response itself, and that reduced proliferation is a core feature of cellular adaptation for survival through changing environments and on a longer timescale, through evolution.

## Materials and methods

### Cell culture

MCF10A cells were maintained in DMEM/F12 (Gibco, Waltham, MA), supplemented with 5% horse serum (Gibco), 20 ng/mL epidermal growth factor ([EGF] Sigma-Aldrich), 0.5 mg/mL hydrocortisone (Sigma-Aldrich, St. Louis, MO), 100 ng/mL cholera toxin (Sigma-Aldrich), 10 μg/mL insulin (Thermo Fisher, Waltham, MA), and 1× penicillin/streptomycin. For serum starvation media, the horse serum, EGF, and insulin were removed, and 0.3% BSA was added. For live-cell time-lapse imaging, phenol red–free DMEM/F12 (Gibco) was used.

To prepare forced quiescent samples for RNA-seq or validation experiments, cells were seeded at a density of 200,000 cells per well in a six-well dish, or 2,000 cells per well in a 96-well dish. Cell number was increased 2-fold for serum starvation, Meki, and CDK4/6i conditions and by 10-fold for the contact inhibition condition. All forced quiescence treatments lasted 48 h. To test the reversibility of the quiescence, cells were released from the treatment into full-growth media for 24 h.

### Stable cell lines

Wild-type and *p21*^−/−^ MCF10A H2B-mTurquoise DHB-Venus cells were described by Spencer and colleagues [10]. MCF10A mCitrine-p21 knock-in cells were described by Moser and colleagues [12]. MCF10A *p53*^−/−^ cells (clone H2PC13) were described by Weiss and colleagues [46], and H2B-mTurquoise and DHB-mVenus were expressed in these cells by means of lentiviral transduction. Lentivirus was used to express Geminin_1–110_-mCherry, H2B-mCherry, or H2B-mTurquoise in MCF10A-mCitrine-p21 cells. Geminin is used as a marker for the start of S phase [18]. FACS was used to sort positive cells.

### Inhibitors and chemicals

The following inhibitors and chemicals were used: CDK4/6 inhibitor, palbociclib (S1116, Selleckchem, Houston, TX) at 1 μM; Mek inhibitor, PD-0325091 (S1036, Selleckchem) at 100 nM; bortezomib (10008822, Cayman Chemical, Ann Arbor, MI) at 1 μM; nutlin-3 (10004372, Cayman Chemical); salubrinal (14735, Cayman Chemical); ganetespib (STA-9090, Selleckchem); gemcitabine (G6423, Sigma); and hydrogen peroxide (5240–05, Maron), at indicated concentrations.

### RNA-seq

Five biological replicates of the Geminin^low^/p21^high^ and Geminin^low^/p21^low^ subpopulations were collected for each clone by FACS. Two biological replicates of the forced-quiescence cells were collected for each clone after treatment for 48 h. In each sample, a cell pellet of 100,000 cells was snap-frozen and stored at −80 °C before library construction.

Total RNA was extracted from each sample using Quick-RNA MicroPrep kit from Zymo (Irvine, CA). The Lexogen RiboCop rRNA Depletion Kit was used for ribosomal RNA removal and the Lexogen SENSE total RNA kit was used to prepare paired-end, stranded libraries. The libraries were pooled at equal amounts using the concentrations measured on a Qubit Fluorometer (Thermo Fisher) and sequenced on an Illumina NextSeq sequencer with 1% PhiX spike-in.

Overall sequence quality was examined using FastQC version 0.11.2. Adaptor sequences were clipped using Trimmomatic version 0.32 [47]. Reads were mapped to the human genome (GRCh38) using HISAT2 version 2.0.3-beta [48]. Mapped fragments were counted using GenomicAlignments package in R [49]. Differential expression analysis was carried out using DESeq2 version 1.10.1 [50], with a negative binomial-generalized linear model including batch and cell clone factor in the design. Genes with an adjusted *p*-value less than 0.05 were considered to be differentially expressed.

GO analysis was carried out using g:Profiler [51], with significance threshold at *p* < 0.01. Pathway analysis was carried out using GAGE R package [52], with significance threshold at *p* < 0.05. The UpSetR plot was generated using UpSetR R package [53].

### Antibodies and siRNA

The following antibodies and siRNA were used: anti–phospho-Rb Ser807/811 (CST, Danvers, MA, production number 8516) at 1:1,000 for western blots and at 1:250 for IF; anti-p21 (CST, 2947) at 1:1,000 for western blots and at 1:250 for IF; anti-eIF2α phospho-Ser51 (CST, 3398) at 1:1,000; anti-eIF2α (CST, 5324) at 1:1,000; anti-p53 (Santa Cruz Biotechnology, Dallas, TX, sc-126) at 1:1,000; anti-Lamp1 (CST, 9091) at 1:1,000; anti-Pink1 (CST, 6946) at 1:1,000; anti-GAPDH (abcam, Cambridge, MA, ab9485) at 1:2,000; anti-tubulin (CST, 86298) at 1:2,000; anti-PKR (CST, 12297) at 1:2,000; anti-PERK (CST, 3192) at 1:1,000; anti-GCN2 (CST, 3302) at 1:1,000; anti-rabbit IgG, HRP-linked (CST, 7074) at 1:2,000; anti-mouse IgG, HRP-linked (CST, 7076) at 1:2,000; goat anti-rabbit and goat anti-mouse 800 (Azure Biosystems, Dublin, CA) at 1:5,000; and Alexa Fluor-647 secondary antibody (Thermo Fisher) at 1:500.

siRNA oligos were synthesized by IDT (San Jose, CA): EIF2AK1 (hs.Ri.EIF2AK1.13.3); EIF2AK2 (hs.Ri.EIF2AK2.13.2); EIF2AK3 (hs.Ri.EIF2AK3.13.3); EIF2AK4 (hs.Ri.EIF2AK4.13.2), and negative control DsiRNA (51-01-14-04). siRNA transfection was carried out using DharmaFECT 1 (Dharmacon, Chicago, IL) following the manufacturer’s instruction. Cells were fixed 24 or 48 h after transfection.

### Live-cell imaging, IF, and RNA FISH

Cells were plated on a 96-well plate (Cellvis, Mountain View, CA, P96-1.5H-N) coated with collagen (Advanced BioMatrix, Carlsbad, CA, #5015) 24 h prior to the start of imaging, at a density such that cells were sub-confluent throughout the imaging period. Cells were imaged on either an ImageXpress Micro XLS wide-field microscope (Molecular Devices, San Jose, CA) with a 10× 0.45 NA objective or on a Nikon Inverted Microscope Eclipse Ti-E PFS (Nikon, Japan) with a 10× 0.45 NA objective with appropriate filter sets. Images were taken by a Zyla 5.5 sCMOS camera (Andor Technology, UK) or an ORCA-Flash 4.0 CMOS camera (Hamamatsu, Japan) at the frequency of 1 frame per 12 min. During the imaging, cells were kept in a humidified, 37 °C chamber at 5% CO_2_. Total light exposure time for each time point was kept under 350 ms. For experiments involving drug treatments, cells were first imaged without drug for 16–24 h; the movie was then paused for 1–5 min and drugs were added by exchanging 50% of the media in each well with media containing a 2× drug concentration. Cells were then imaged for an additional 24 to 48 h.

In experiments in which live-cell imaging was followed by IF or RNA FISH, cells were fixed immediately after live-cell imaging by incubation in 4% paraformaldehyde for 15 min. In EdU, OPP, or EU incorporation experiments, cells were incubated in media containing 10 μM EdU for 15 min, 20 μM OPP for 24 min, or 1 mM EU for 24 min and then fixed and processed according to the manufacturer’s instructions (ThermoFisher C10340, C10458, and C10330).

For IF, cells were incubated with a blocking/permeabilization buffer (3% BSA, 0.1% Triton X-100) for 1 h at room temperature. Primary antibody staining was carried out overnight at 4 °C in the blocking buffer and visualized using secondary antibodies conjugated to Alexa Fluor 647. RNA FISH was carried out using the ViewRNA ISH Cell Assay kit (Thermo Fisher) following the manufacturer’s instruction. The FISH probes used were as follows: PGK1 (VA1-12352); CCNE1 (VA6-3167995); E2F1 (VA6-3168356-VC); CCNA2 (VA6-15304); and CCNB1 (VA6-16942).

### Image processing and cell tracking

Image processing and cell tracking were performed using a published pipeline [54] available at https://github.com/scappell/Cell_tracking. In brief, camera dark noise was subtracted from the raw images, which were then divided by the illumination bias. Dark noise was measured by a blank image taken with light power off. The illumination bias of each fluorescent channel was estimated by the averaged cell-free contour of all images in that channel. Log-transformed H2B-mTurquoise images were then convolved with a rotationally symmetric Laplacian of Gaussian filter, and objects were defined as contiguous pixels exceeding a threshold filter score. Segmented cell nuclei were tracked by screening the nearest future neighbor. The background of each image was subtracted using top-hat filtering. Mean nuclear intensities were measured by averaging the background-subtracted pixel intensities in each nucleus as defined by a segmented nuclear mask. CDK2 activity was calculated as the ratio of cytoplasmic to nuclear median sensor fluorescence, with the cytoplasmic component measured in a four-pixel-wide cytoplasmic ring outside of the nuclear mask.

In experiments in which IF or FISH signals were matched back to the live-cell imaging, the IF/FISH images were mapped to the last frame of the corresponding live-cell image using nearest neighbor screening after jitter correction. FISH intensity was quantified as median pixel value in a four-pixel-wide cytoplasmic ring outside of the nuclear mask.

To segment 53BP1 nuclear bodies and γH2AX foci, images of 53BP1 and γH2AX staining were top-hat filtered to remove nuclear background before thresholding. A segmented focus was assigned to a nucleus if they shared at least one pixel. Cells with at least one 53BP1 nuclear body were classified as 53BP1 n.b.^+^.

### Single-cell analysis

Each daughter cell was classified as CDK2^inc^, CDK2^low^, or CDK2^emerge^ as follows: CDK2^inc^ cells have CDK2 activity greater than 0.5 at 3 h after anaphase; CDK2^low^ cells have CDK2 activity less than or equal to 0.5 at 3 h after anaphase and stay below 0.5 for the rest of the movie; and CDK2^emerge^ cells have CDK2 activity less than 0.5 at 3 h after anaphase and rise above 0.5 later in the movie.

The R-point was determined as the time CDK2 activity first begins to rise. Computationally, this involves calculating slopes of CDK2 activity using windows of 6–10 time points, and then maximizing a linear function for time since mitosis, CDK2 activity, and CDK2 slope (long times since mitosis, low CDK2 activity, and high CDK2 slope). The rise point of CDK2 activity was manually verified for each cell.

To determine low/high thresholds for staining intensity of phospho-Rb, p21 protein, and E2F1/CCNE1 mRNA, the Otsu method was used to compute a global threshold on intensity distributions of all cells in the same experiment [29].

Lineage survival is defined as follows: each cell at the frame of drug addition is considered as a lineage; the end of a lineage is defined as the time of cell death of the last cell in the lineage. For example, if a cell divides once after drug addition, the end of this lineage is the time that both daughter cells have died. The times of cell death were manually determined by condensation of the nuclear marker H2B without cell division.

## Supporting information

S1 Fig Validation of the slow-cycling subpopulation. (A) Percentage of cells with 53BP1 n.b. in WT MCF10A and MCF10A expressing H2B-mTurquoise and DHB-mVenus. Error bars indicate standard error of the mean. *n* = 6 wells for each cell line. (B) IMT distribution of wild-type MCF10A imaged with phase contrast only (left), H2B-mTurquoise/DHB-mVenus MCF10A imaged with phase contrast only (middle), or H2B-mTurquoise/DHB-mVenus MCF10A imaged with phase contrast plus fluorescent exposure used in other experiments in this paper (right). (C) Normalized IMT distributions for the three conditions in B, with the area under the curves equal to 1. (D) Correlation coefficient (*R*) between G1–S–G2–M length and IMT, and between G0 length and IMT, in populations with IMT longer than the cutoff indicated along the x-axis. (E) Sorted p21^high^ cells have high levels of p21 and low levels of phospho-Rb S807/811, as measured by western blot immediately after sorting. (F) Density scatterplot of mCherry-Geminin_1–110_ intensity versus DNA content. (G) Density scatterplot shows the distribution of mCitrine-p21 and mCherry-Geminin_1–110_ intensity in parental WT (left) and mCitrine-p21 mCherry-Geminin_1–110_ cells (right). Blue and red boxes highlight collection gates for p21^low^ and p21^high^ subpopulations, respectively. (H) Distribution of p21 IF staining intensity shows that both mCitrine-p21 clones express similar level of p21. Underlying data for this figure can be found in the BioStudies database under accession number S-BSST231. IF, immunofluorescence; IMT, intermitotic time; n.b., nuclear body; WT, wild-type.
(PDF)

S2 Fig Protein levels of p53, LAMP1, and PINK1 in quiescence cells. (A) Western blot of p53, LAMP1, and PINK1 in sorted p21^high^ cells, sorted p21^low^ cells, and forced-quiescence cells. Quantification of blots are shown to the right, with proteins level of interest first normalized by GAPDH levels and then normalized to those in the p21^low^ sample or the control sample of the same clone. Error bars indicate standard error of the mean, *n* = 3 repeats. (B) Western blot of p53 and LAMP1 in sorted p21^high^ cells and p21^low^ cells for four different mCitrine-p21 knock-in clones. Clone 2E2 has one knock-in allele and one wild-type allele; clone 3B6 has one mCitrine knock-in allele and one p21 knockout allele. Quantification of blots are shown at the bottom, with proteins level of interest first normalized by GAPDH levels. (C) Percentage of cells with 53BP1 n.b. or γH2AX foci in phospho-Rb^low^ and phospho-Rb^high^ subpopulations. Underlying data for this figure can be found in the BioStudies database under accession number S-BSST231. n.b., nuclear body.
(PDF)

S3 Fig Validation of the forced quiescence populations. (A) Representative images of control proliferating cells, serum-starved cells, contact-inhibited cells, and cells treated with CDK4/6 inhibitor or Mek inhibitor. Scale bar, 400 μm. (B) Column 1–3, density scatterplots of EdU incorporation versus DNA content. Percentage of EdU-positive cells is indicated in the upper right corner of each plot. Column 1, control cells; Column 2, cells at the end of 48-h treatments; Column 3, cells released from 48-h treatments into full-growth conditions for 24 h; Column 4, distribution of phospho-Rb under control, forced-quiescence, and released conditions. Underlying data for this figure can be found in the BioStudies database under accession number S-BSST231. EdU, 5-ethynyl deoxyuridine.
(PDF)

S4 Fig Related to Fig 2. (A) PCA analysis of all samples for both mCitrine-p21 knock-in clones, 2e2 and 3b6. For simplicity, two out of five biological replicates for spontaneous quiescence samples were plotted. Control samples are untreated, unsorted cells. The two clones are separated by PC2, indicating clonal effects. However, the relative positioning of the five quiescence conditions within each clone is consistent between the two clones. Hence, condition differences can be separated from clonal differences. (B) UpSetR plot shows the intersection and difference of genes differentially regulated in five forms of quiescence. Red highlights the gene set uniquely up-regulated in spontaneous quiescence (287 genes) or the gene set up-regulated in all five forms of quiescence (70 genes); blue highlights the gene set uniquely down-regulated in spontaneous quiescence (168 genes) or the gene set universally down-regulated in all five forms of quiescence (128 genes). Underlying data for this figure can be found in the GEO database under accession number GSE122927. PC2, principal component 2; PCA, principal component analysis.
(PDF)

S5 Fig Box plot of mRNA level in p21^high^ versus p21^low^ cells measured by RNA-seq for each clone corresponding to column 3 in Fig 3A–3F. Underlying data for this figure can be found in the GEO database under accession number GSE122927. RNA-seq, RNA sequencing.
(PDF)

S6 Fig Related to Fig 4. (A) Bar plot shows differential expression of ATF4 transcriptional targets in five forms of quiescence. (B) Western blot shows that our ATF4 antibody cannot detect any specific signal in unperturbed cells, although it shows strong staining in samples in which the ISR is activated by proteasome inhibition-induced amino acid depletion (bortezomib treatment for 4 h). (C) Hoechst and EU images show lack of transcription in mitosis. Red stars mark metaphase and anaphase cells that are known to suppress transcription, thereby demonstrating specificity of the EU assay. Blue stars mark cells in which chromatin is starting to decondense and transcription is turning back on. (D) Density plot of phospho-Rb S807/811 intensity after control siRNA treatment or knockdown of the four eIF2α kinases. (E) Validation of knockdown in D by western blotting for PKR, PERK, and GCN2. Top, a representative blot; the star in the GCN2 blot marks a nonspecific band. Bottom, quantification of protein level with normalization to tubulin (mean ± standard deviation of two repeats). Underlying data for this figure can be found in the BioStudies database under accession number S-BSST231. EU, 5-ethynyl uridine; ISR, integrated stress response; siRNA, small interfering RNA.
(PDF)

S7 Fig Related to Fig 5, lineage survival under lower doses of stress. (A) Lineage survival of cells that are CDK2^low^ at the time of drug addition (red), cells that are CDK2^inc^ at the time of drug addition but converted to CDK2^low^ later (yellow), or cells that are CDK2^inc^ until their death or the end of the imaging period (blue). The percentage of each cell category is indicated to the right. (B) Lineage survival of wild-type versus *p21*^−/−^ cells. Underlying data for this figure can be found in the BioStudies database under accession number S-BSST231.
(PDF)

S1 Table Relative expression of all genes in the human genome across five quiescence conditions. For spontaneous quiescence, the differential expression is calculated as p21^high^ cells versus p21^low^ cells. For other forms of quiescence, the comparison is perturbed cells versus control cells.
(XLSB)

S2 Table GO terms that are enriched in genes up-regulated or down-regulated in p21^high^ versus p21^low^ cells. GO, Gene Ontology.
(XLSX)

S3 Table List of genes universally up- or down-regulated in all five forms of quiescence. (XLSX)

S4 Table Overlap between S3 Table and a published “fibroblast quiescence program” [14]. (XLSX)

S5 Table List of DREAM and p53 targets used in Fig 2D. DREAM, dimerization partner, RB-like, E2F4, and multi-vulval class B.
(XLSX)

S1 Movie Survival of wild-type cells in control condition. Wild-type cells expressing CDK2 activity sensor (DHB-mVenus) treated with DMSO at the start of the movie were imaged every 12 min for 50 h.
(AVI)

S2 Movie Survival of *p21*^−/−^ cells in control condition. *p21*^−/−^ cells expressing CDK2 activity sensor (DHB-mVenus) treated with DMSO at the start of the movie were imaged every 12 min for 50 h.
(AVI)

S3 Movie Survival of wild-type cells in unfolded protein stress. Wild-type cells expressing CDK2 activity sensor (DHB-mVenus) treated with 1 μM ganetespib at the start of the movie were imaged every 12 min for 50 h.
(AVI)

S4 Movie Survival of *p21*^−/−^ cells in unfolded protein stress. *p21*^−/−^ cells expressing CDK2 activity sensor (DHB-mVenus) treated with 1 μM ganetespib at the start of the movie were imaged every 12 min for 50 h.
(AVI)

## Abbreviations

a.u.: arbitrary unit
ABC: ATP-binding cassette
CDK: cyclin-dependent kinase
ChIP-Seq: chromatin immunoprecipitation sequencing
CuAAC: copper-catalyzed alkyne-azide cycloaddition
DREAM: dimerization partner, RB-like, E2F4, and multi-vulval class B
ECM: extracellular matrix
EdU: 5-ethynyl deoxyuridine
EGF: epidermal growth factor
EU: 5-ethynyl uridine
FACS: fluorescence-activated cell sorting
FUCCI: fluorescence ubiquitination cell cycle indicator
GO: Gene Ontology
GRO-Seq: global run-on sequencing
IF: immunofluorescence
IMT: intermitotic time
ISR: integrated stress response
MAPK: mitogen-activated protein kinase
MCM: minichromosome maintenance protein complex
MMB-FOXM1: MuvB complex with B-Myb or FOXM1
n.b.: nuclear body
OPP: O-propargyl-puromycin
PCA: principal component analysis
RNA FISH: RNA fluorescence in situ hybridization
RNA-seq: RNA sequencing
R-point: Restriction Point
siRNA: small interfering RNA
TPM: transcripts per million
YFP: yellow fluorescent protein
