# Supplementary figures and images for: Spontaneously slow-cycling subpopulations of human cells originate from activation of stress-response pathways
Source: PLoS Biol. 2019 Mar 13;17(3):e3000178. doi: 10.1371/journal.pbio.3000178 (PMC6433297; doi:10.1371/journal.pbio.3000178)

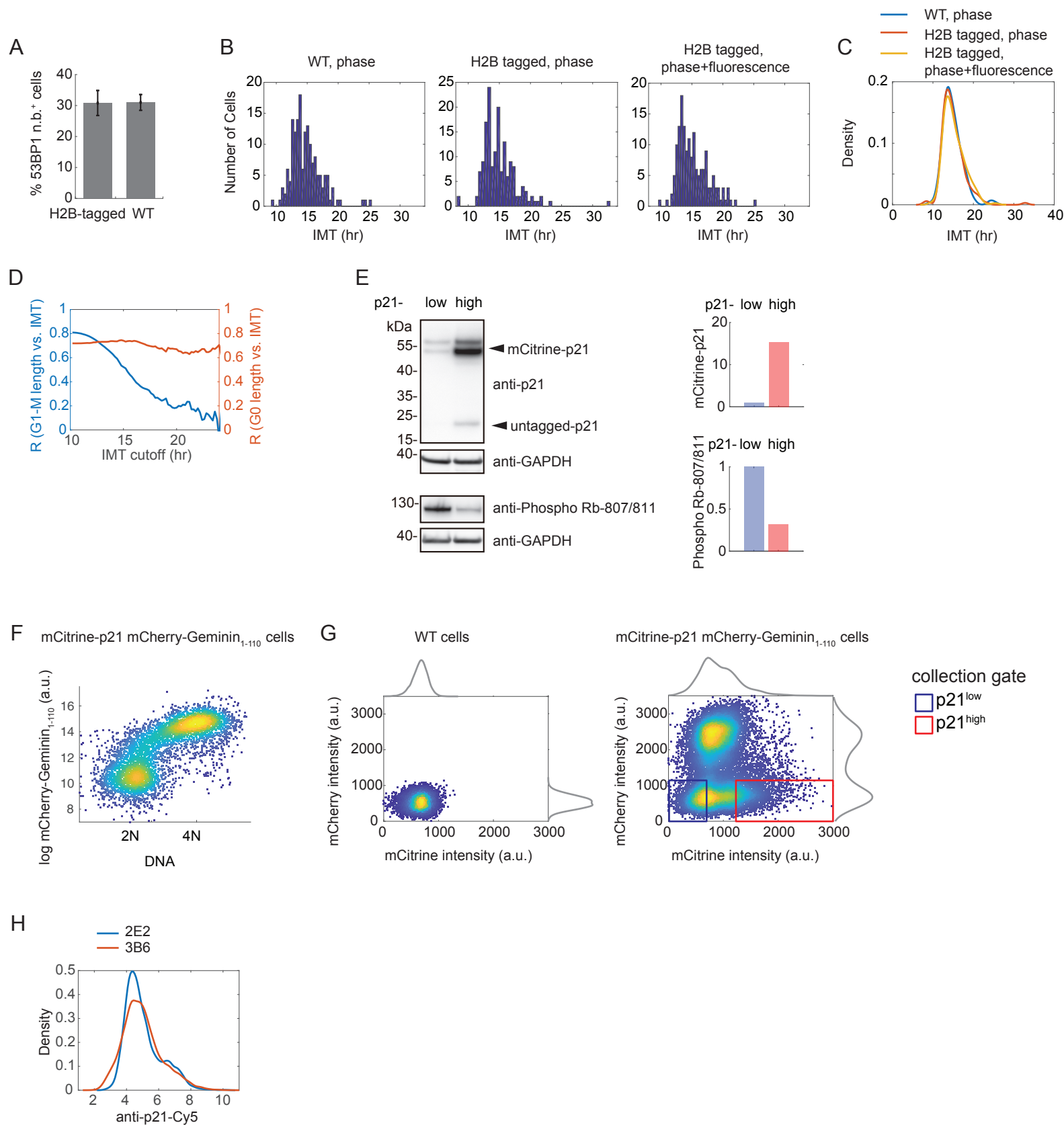

**Figure S1**

Supplement: S1 Fig — (A) Percentage of cells with 53BP1 n.b. in WT MCF10A and MCF10A expressing H2B-mTurquoise and DHB-mVenus. Error bars indicate standard error of the mean. n = 6 wells for each cell line. (B) IMT distribution of wild-type MCF10A imaged with phase contrast only (left), H2B-mTurquoise/DHB-mVenus MCF10A imaged with phase contrast only (middle), or H2B-mTurquoise/DHB-mVenus MCF10A imaged with phase contrast plus fluorescent exposure used in other experiments in this paper (right). (C) Normalized IMT distributions for the three conditions in B, with the area under the curves equal to 1. (D) Correlation coefficient (R) between G1–S–G2–M length and IMT, and between G0 length and IMT, in populations with IMT longer than the cutoff indicated along the x-axis. (E) Sorted p21high cells have high levels of p21 and low levels of phospho-Rb S807/811, as measured by western blot immediately after sorting. (F) Density scatterplot of mCherry-Geminin1–110 intensity versus DNA content. (G) Density scatterplot shows the distribution of mCitrine-p21 and mCherry-Geminin1–110 intensity in parental WT (left) and mCitrine-p21 mCherry-Geminin1–110 cells (right). Blue and red boxes highlight collection gates for p21low and p21high subpopulations, respectively. (H) Distribution of p21 IF staining intensity shows that both mCitrine-p21 clones express similar level of p21. Underlying data for this figure can be found in the BioStudies database under accession number S-BSST231. IF, immunofluorescence; IMT, intermitotic time; n.b., nuclear body; WT, wild-type. (PDF) [file pbio.3000178.s001.pdf]

A

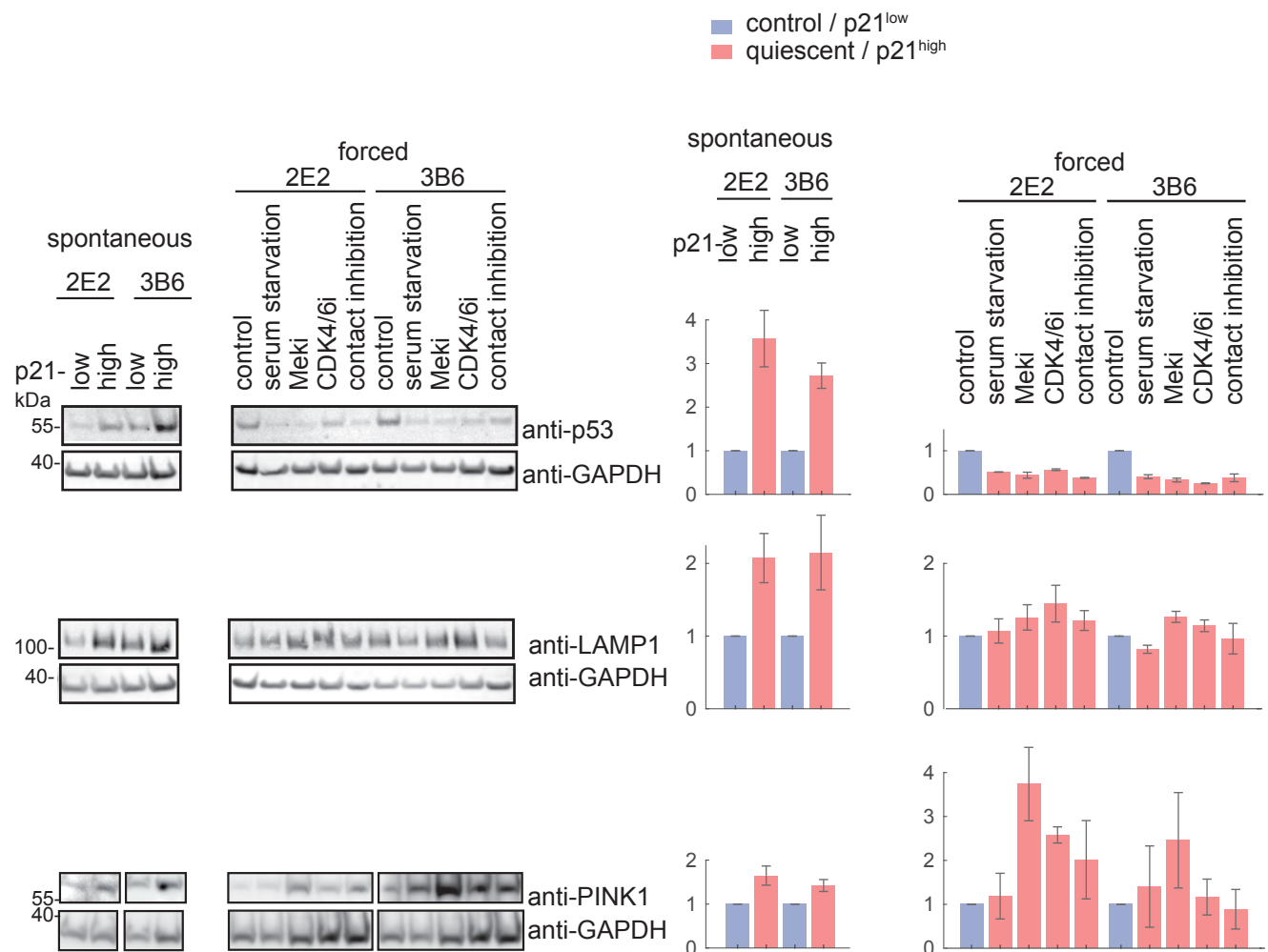

B

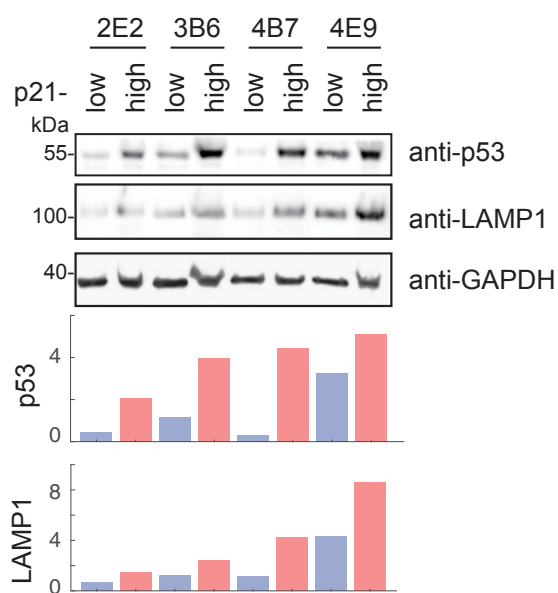

C

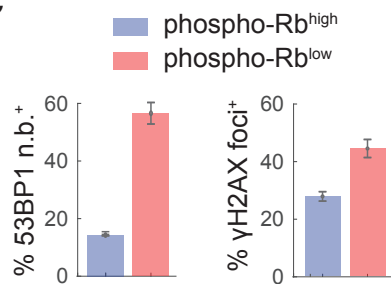

Figure S2

Supplement: S2 Fig — (A) Western blot of p53, LAMP1, and PINK1 in sorted p21high cells, sorted p21low cells, and forced-quiescence cells. Quantification of blots are shown to the right, with proteins level of interest first normalized by GAPDH levels and then normalized to those in the p21low sample or the control sample of the same clone. Error bars indicate standard error of the mean, n = 3 repeats. (B) Western blot of p53 and LAMP1 in sorted p21high cells and p21low cells for four different mCitrine-p21 knock-in clones. Clone 2E2 has one knock-in allele and one wild-type allele; clone 3B6 has one mCitrine knock-in allele and one p21 knockout allele. Quantification of blots are shown at the bottom, with proteins level of interest first normalized by GAPDH levels. (C) Percentage of cells with 53BP1 n.b. or γH2AX foci in phospho-Rblow and phospho-Rbhigh subpopulations. Underlying data for this figure can be found in the BioStudies database under accession number S-BSST231. n.b., nuclear body. (PDF) [file pbio.3000178.s002.pdf]

A

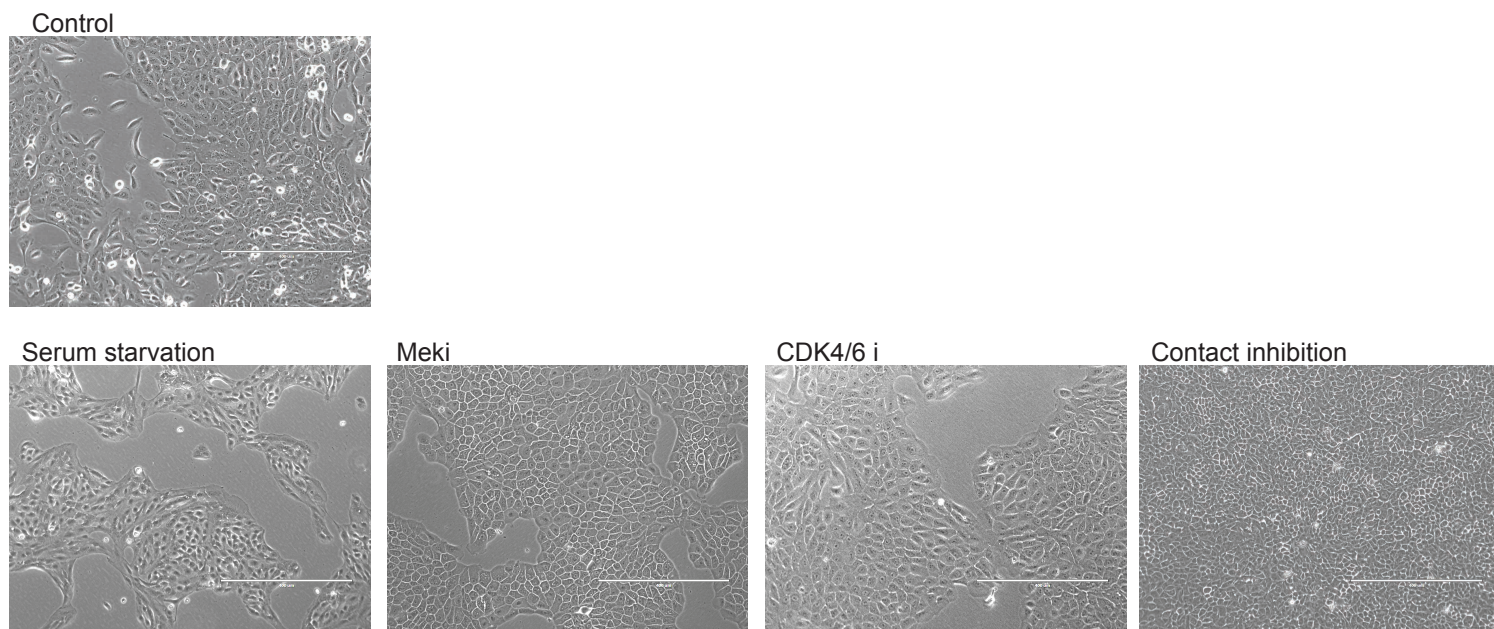

B

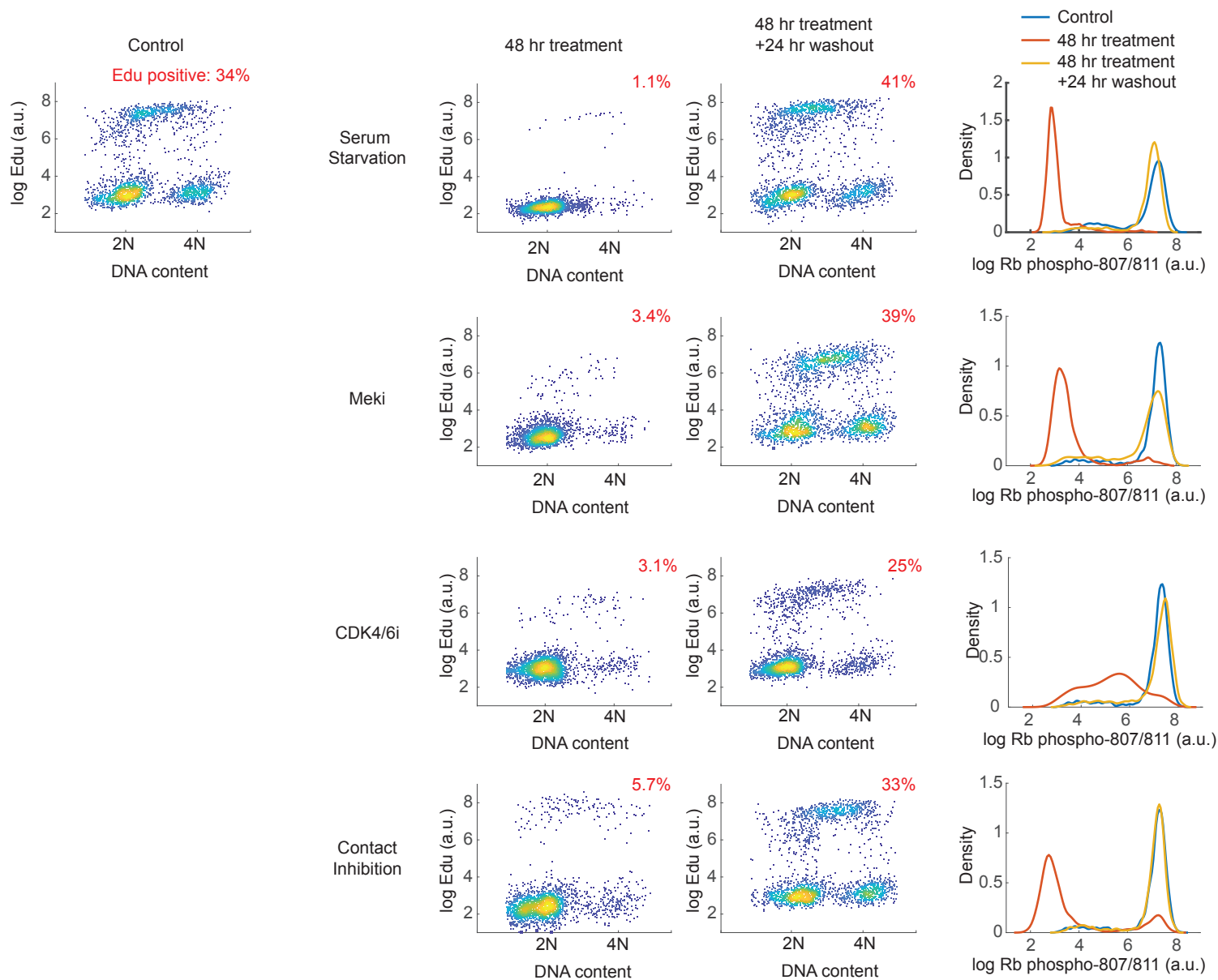

Figure S3

Supplement: S3 Fig — (A) Representative images of control proliferating cells, serum-starved cells, contact-inhibited cells, and cells treated with CDK4/6 inhibitor or Mek inhibitor. Scale bar, 400 μm. (B) Column 1–3, density scatterplots of EdU incorporation versus DNA content. Percentage of EdU-positive cells is indicated in the upper right corner of each plot. Column 1, control cells; Column 2, cells at the end of 48-h treatments; Column 3, cells released from 48-h treatments into full-growth conditions for 24 h; Column 4, distribution of phospho-Rb under control, forced-quiescence, and released conditions. Underlying data for this figure can be found in the BioStudies database under accession number S-BSST231. EdU, 5-ethynyl deoxyuridine. (PDF) [file pbio.3000178.s003.pdf]

A

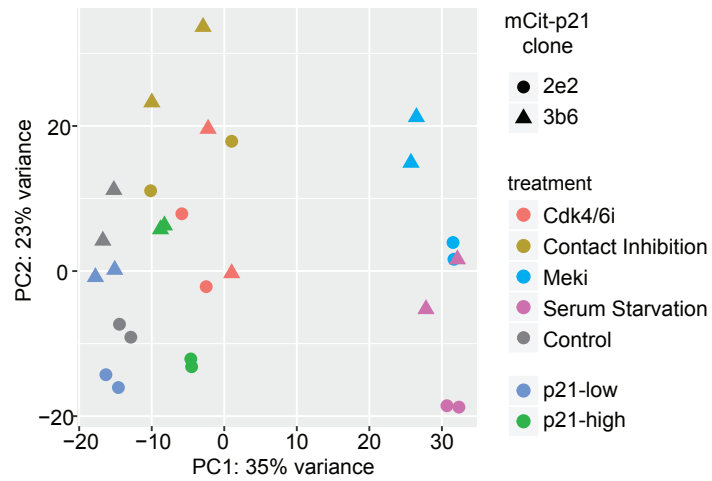

B

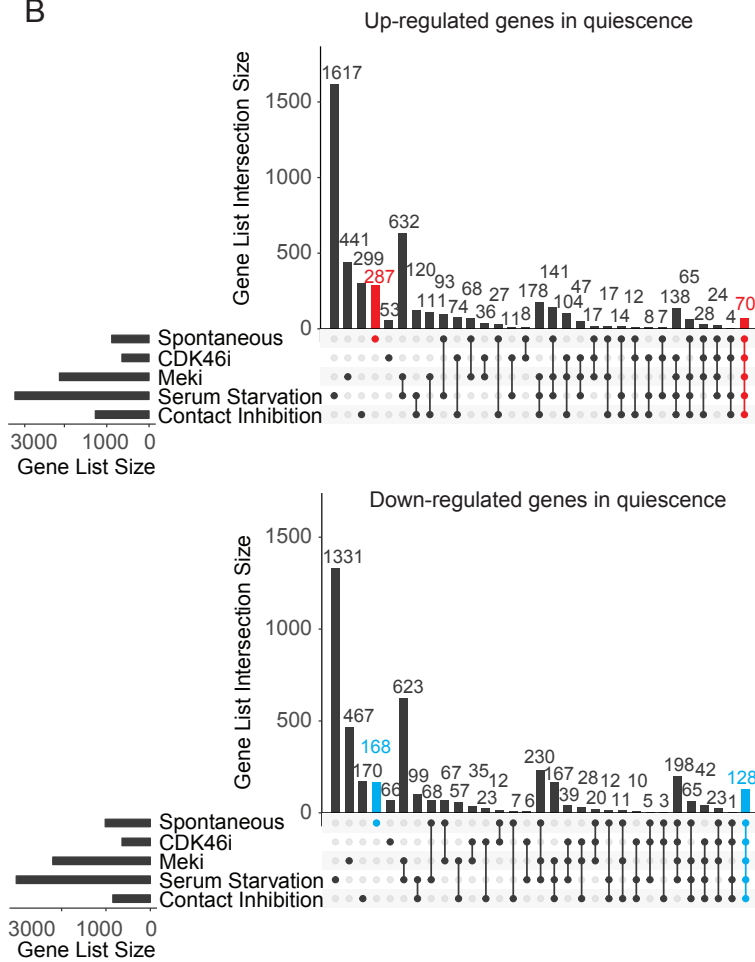

Figure S4

Supplement: S4 Fig — (A) PCA analysis of all samples for both mCitrine-p21 knock-in clones, 2e2 and 3b6. For simplicity, two out of five biological replicates for spontaneous quiescence samples were plotted. Control samples are untreated, unsorted cells. The two clones are separated by PC2, indicating clonal effects. However, the relative positioning of the five quiescence conditions within each clone is consistent between the two clones. Hence, condition differences can be separated from clonal differences. (B) UpSetR plot shows the intersection and difference of genes differentially regulated in five forms of quiescence. Red highlights the gene set uniquely up-regulated in spontaneous quiescence (287 genes) or the gene set up-regulated in all five forms of quiescence (70 genes); blue highlights the gene set uniquely down-regulated in spontaneous quiescence (168 genes) or the gene set universally down-regulated in all five forms of quiescence (128 genes). Underlying data for this figure can be found in the GEO database under accession number GSE122927. PC2, principal component 2; PCA, principal component analysis. (PDF) [file pbio.3000178.s004.pdf]

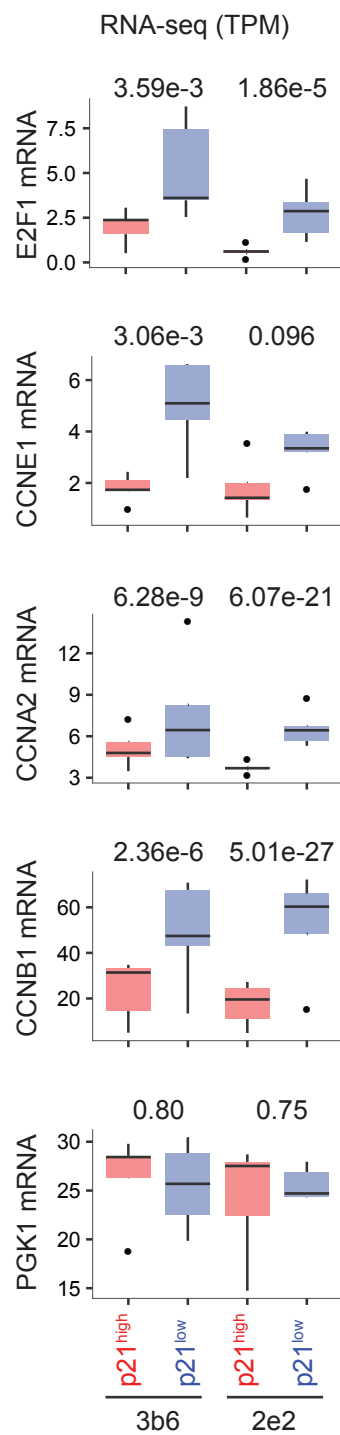

Figure S5

Supplement: S5 Fig — Underlying data for this figure can be found in the GEO database under accession number GSE122927. RNA-seq, RNA sequencing. (PDF) [file pbio.3000178.s005.pdf]

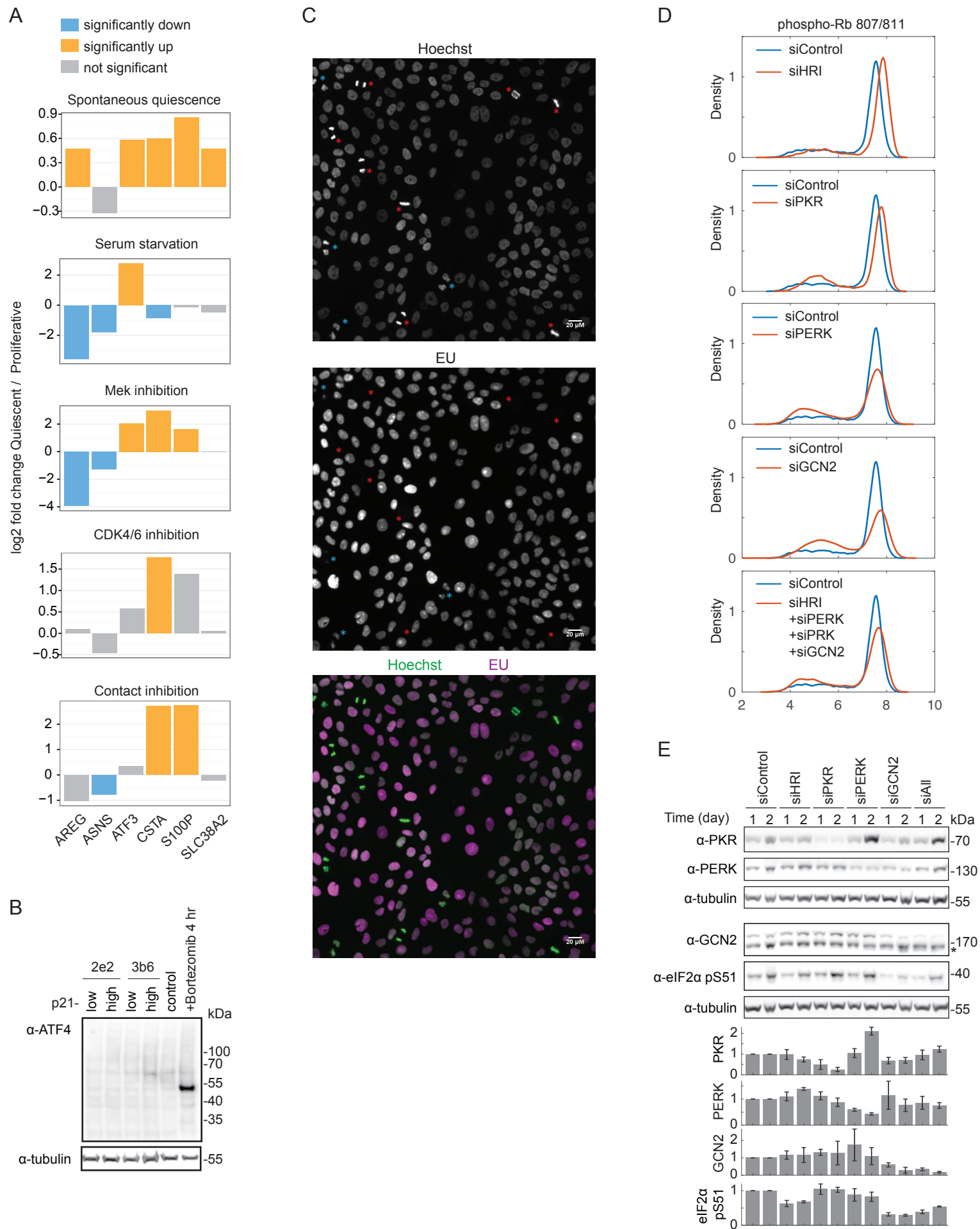

**Figure S6**

Supplement: S6 Fig — (A) Bar plot shows differential expression of ATF4 transcriptional targets in five forms of quiescence. (B) Western blot shows that our ATF4 antibody cannot detect any specific signal in unperturbed cells, although it shows strong staining in samples in which the ISR is activated by proteasome inhibition-induced amino acid depletion (bortezomib treatment for 4 h). (C) Hoechst and EU images show lack of transcription in mitosis. Red stars mark metaphase and anaphase cells that are known to suppress transcription, thereby demonstrating specificity of the EU assay. Blue stars mark cells in which chromatin is starting to decondense and transcription is turning back on. (D) Density plot of phospho-Rb S807/811 intensity after control siRNA treatment or knockdown of the four eIF2α kinases. (E) Validation of knockdown in D by western blotting for PKR, PERK, and GCN2. Top, a representative blot; the star in the GCN2 blot marks a nonspecific band. Bottom, quantification of protein level with normalization to tubulin (mean ± standard deviation of two repeats). Underlying data for this figure can be found in the BioStudies database under accession number S-BSST231. EU, 5-ethynyl uridine; ISR, integrated stress response; siRNA, small interfering RNA. (PDF) [file pbio.3000178.s006.pdf]

A

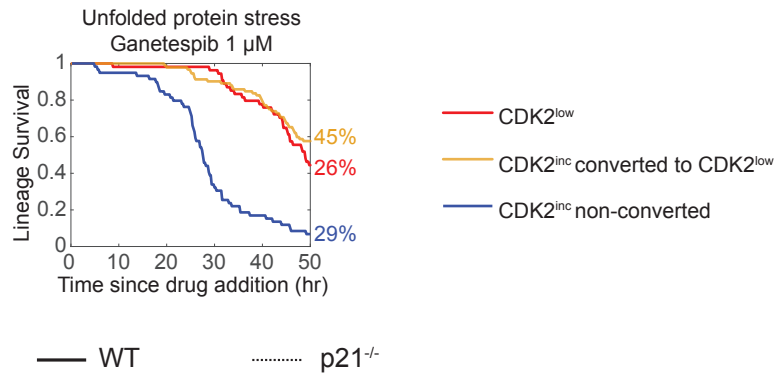

B

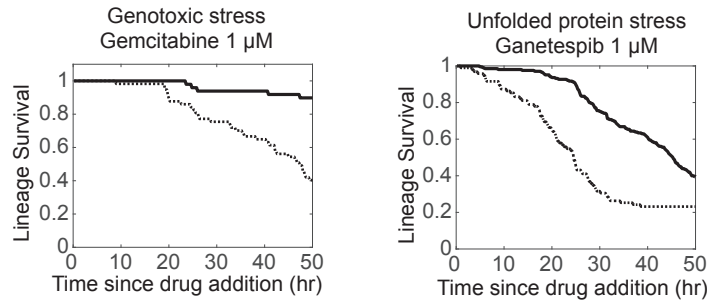

Figure S7

Supplement: S7 Fig — (A) Lineage survival of cells that are CDK2low at the time of drug addition (red), cells that are CDK2inc at the time of drug addition but converted to CDK2low later (yellow), or cells that are CDK2inc until their death or the end of the imaging period (blue). The percentage of each cell category is indicated to the right. (B) Lineage survival of wild-type versus p21−/− cells. Underlying data for this figure can be found in the BioStudies database under accession number S-BSST231. (PDF) [file pbio.3000178.s007.pdf]
